# Supplementary material for: A 250 plastome phylogeny of the grass family (Poaceae): topological support under different data partitions
Source: PeerJ. 2018 Feb 2;6:e4299. doi: 10.7717/peerj.4299 (PMC5798404; doi:10.7717/peerj.4299)
Supplement: Supplemental Information 2 — Letters identifying each of the 14 trees correspond to the data partitions as identified in Table 2. (A) rbcL, ndhF, matK, and trnK intron, including gapped sites and positively selected sites. (B) rbcL, ndhF, matK, and trnK intron, excluding gapped sites and including positively selected sites. (C) rbcL, ndhF, matK, and trnK intron, including gapped sites and excluding positively selected sites. (D) rbcL, ndhF, matK, and trnK intron, excluding gapped sites and positively selected sites. (E) Plastome coding regions, including gapped sites and positively selected sites. (F) Plastome coding regions, excluding gapped sites and including positively selected sites. (G) Plastome coding regions, including gapped sites and excluding positively selected sites. (H) Plastome coding regions, excluding gapped sites and positively selected sites. (Q) Plastome noncoding regions, including gapped sites. (R) Plastome noncoding regions, excluding gapped sites. (W) Complete plastomes, including gapped sites and positively selected sites. (X) Complete plastomes, excluding gapped sites and including positively selected sites. (Y) Complete plastomes, including gapped sites and excluding positively selected sites. (Z) Complete plastomes, excluding gapped sites and positively selected sites. In each subfigure, the bootstrap tree is on the left, with bootstrap proportions indicated along branches, and the best ML tree is on the right. [file peerj-06-4299-s002.pdf]

A

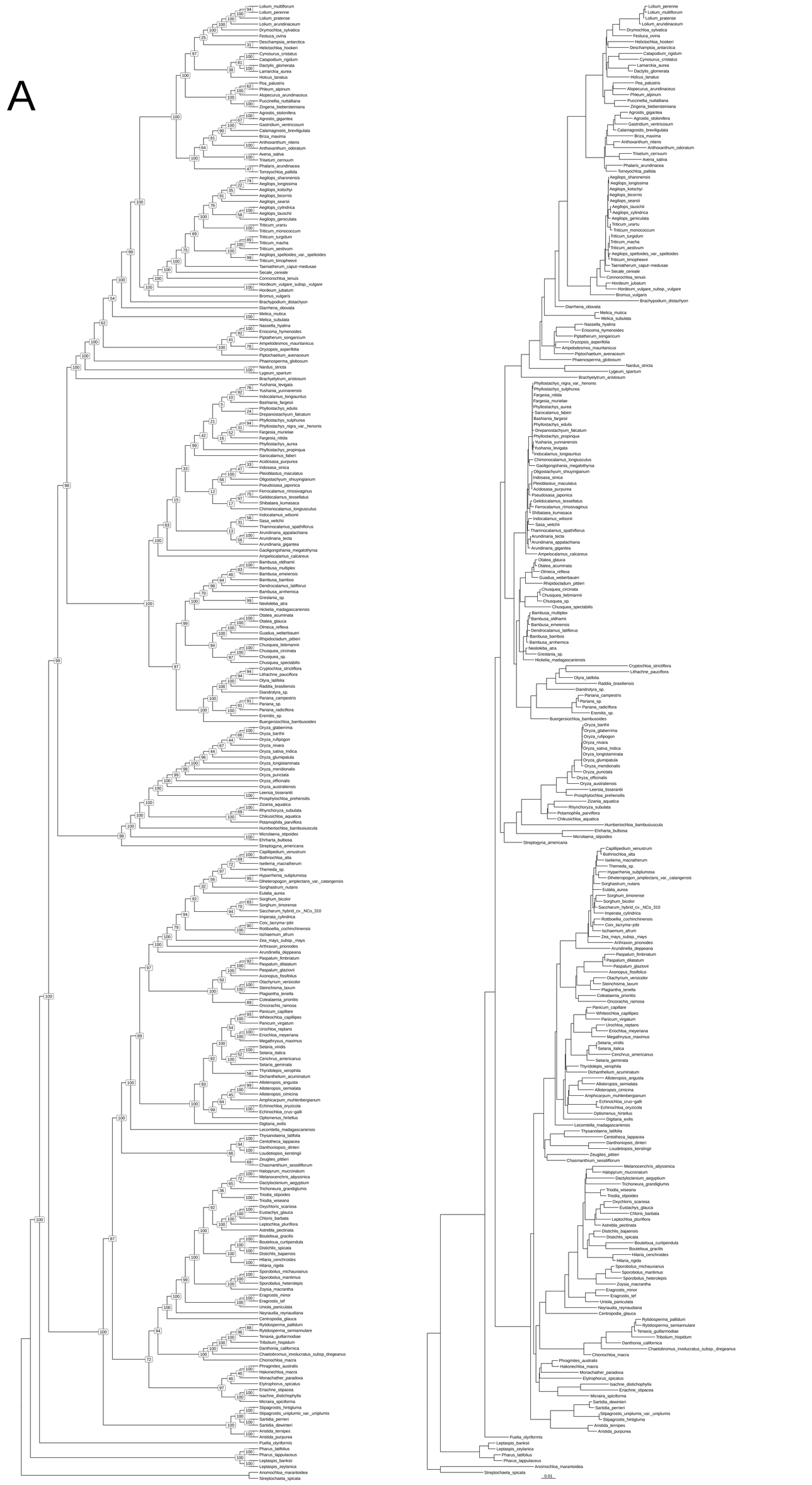
Lolium_multiflorum
Lolium_perenne
Lolium_pratense
Lolium_arundinaceum
Drymochloa_sylvatica
Festuca_ovina
Deschampsia_antarctica
Helictochloa_hookeri
Cynosurus_cristatus
Catapodium_rigidum
Dactylis_glomerata
Lamarckia_aurea
Holcus_lanatus
Poa_palustris
Phleum_alpinum
Alopecurus_arundinaceus
Puccinellia_nuttalliana
Zingeria_biebersteiniana
Agrostis_stolonifera
Agrostis_gigantea
Gastridium_ventricosum
Calamagrostis_breviligulata
Briza_maxima
Anthoxanthum_nitens
Anthoxanthum_odoratum
Avena_sativa
Trisetum_cernuum
Phalaris_arundinacea
Torreyochloa_pallida
Aegilops_sharonensis
Aegilops_longissima
Aegilops_kotschyi
Aegilops_bicornis
Aegilops_searsii
Aegilops_cylindrica
Aegilops_tauschii
Aegilops_geniculata
Triticum_urartu
Triticum_monococcum
Triticum_turgidum
Triticum_macha
Triticum_aestivum
Aegilops_speltoides_var._speltoides
Triticum_timopheevii
Taeniatherum_caput−medusae
Secale_cereale
Connorochloa_tenuis
Hordeum_vulgare_subsp._vulgare
Hordeum_jubatum
Bromus_vulgaris
Brachypodium_distachyon
Diarrhena_obovata
Melica_mutica
Melica_subulata
Nassella_hyalina
Eriocoma_hymenoides
Piptatherum_songaricum
Ampelodesmos_mauritanicus
Oryzopsis_asperifolia
Piptochaetium_avenaceum
Phaenosperma_globosum
Nardus_stricta
Lygeum_spartum
Brachyelytrum_aristosum
Yushania_levigata
Yushania_yunnanensis
Indocalamus_longiauritus
Bashania_fargesii
Phyllostachys_edulis
Drepanostachyum_falcatum
Phyllostachys_sulphurea
Phyllostachys_nigra_var._henonis
Fargesia_murielae
Fargesia_nitida
Phyllostachys_aurea
Phyllostachys_propinqua
Sarocalamus_faberi
Acidosasa_purpurea
Indosasa_sinica
Pleioblastus_maculatus
Oligostachyum_shiuyinginanum
Pseudosasa_japonica
Ferrocalamus_rimosivaginus
Gelidocalamus_tessellatus
Shibataea_kumasaca
Chimonocalamus_longiusculus
Indocalamus_wilsonii
Sasa_veitchii
Thamnocalamus_spathiflorus
Arundinaria_appalachiana
Arundinaria_tecta
Arundinaria_gigantea
Gaoligongshania_megalothyrsa
Ampelocalamus_calcareus
Bambusa_oldhamii
Bambusa_multiplex
Bambusa_emeiensis
Bambusa_bambos
Dendrocalamus_latiflorus
Bambusa_arnhemica
Greslania_sp.
Neololeba_atra
Hickelia_madagascariensis
Otatea_acuminata
Otatea_glauca
Olmeca_reflexa
Guadua_weberbaueri
Rhipidocladum_pittieri
Chusquea_liebmannii
Chusquea_circinata
Chusquea_sp.
Chusquea_spectabilis
Cryptochloa_strictiflora
Lithachne_pauciflora
Olyra_latifolia
Raddia_brasiliensis
Diandrolyra_sp.
Pariana_campestris
Pariana_sp.
Pariana_radiciflora
Eremitis_sp.
Buergersiochloa_bambusoides
Oryza_glaberrima
Oryza_barthii
Oryza_rufipogon
Oryza_nivara
Oryza_sativa_Indica
Oryza_longistaminata
Oryza_glumipatula
Oryza_meridionalis
Oryza_punctata
Oryza_officinalis
Oryza_australiensis
Leersia_tisserantii
Prosphytochloa_prehensilis
Zizania_aquatica
Rhynchoryza_subulata
Chikusichloa_aquatica
Potamophila_parviflora
Humbertochloa_bambusiuscula
Ehrharta_bulbosa
Microlaena_stipoides
Streptogyna_americana
Capillipedium_venustrum
Bothriochloa_alta
Iseilema_macratherum
Themeda_sp.
Hyparrhenia_subplumosa
Diheteropogon_amplectans_var._catangensis
Sorghastrum_nutans
Eulalia_aurea
Sorghum_bicolor
Sorghum_timorense
Saccharum_hybrid_cv._NCo_310
Imperata_cylindrica
Coix_lacryma−jobi
Rottboellia_cochinchinensis
Ischaemum_afrum
Zea_mays_subsp._mays
Arthraxon_prionodes
Arundinella_deppeana
Paspalum_fimbriatum
Paspalum_dilatatum
Paspalum_glaziovii
Axonopus_fissifolius
Otachyrium_versicolor
Steinchisma_laxum
Plagiantha_tenella
Coleataenia_prionitis
Oncorachis_ramosa
Panicum_capillare
Whiteochloa_capillipes
Panicum_virgatum
Urochloa_reptans
Eriochloa_meyeriana
Megathyrsus_maximus
Setaria_viridis
Setaria_italica
Cenchrus_americanus
Setaria_geminata
Thyridolepis_xerophila
Dichanthelium_acuminatum
Alloteropsis_angusta
Alloteropsis_semialata
Alloteropsis_cimicina
Amphicarpum_muhlenbergianum
Echinochloa_crus−galli
Echinochloa_oryzicola
Oplismenus_hirtellus
Digitaria_exilis
Lecomtella_madagascariensis
Thysanolaena_latifolia
Centotheca_lappacea
Danthoniopsis_dinteri
Loudetiopsis_kerstingii
Zeugites_pittieri
Chasmanthium_sessiliflorum
Melanocenchris_abyssinica
Halopyrum_mucronatum
Dactyloctenium_aegyptium
Trichoneura_grandiglumis
Triodia_wiseana
Triodia_stipoides
Oxychloris_scariosa
Eustachys_glauca
Chloris_barbata
Leptochloa_plurifiora
Astrebla_pectinata
Bouteloua_gracilis
Bouteloua_curtipendula
Distichlis_spicata
Distichlis_bajaensis
Hilaria_cenchroides
Hilaria_rigida
Sporobolus_michauxianus
Sporobolus_maritimus
Sporobolus_heterolepis
Zoysia_macrantha
Eragrostis_minor
Eragrostis_tef
Uniola_paniculata
Neyraudia_reynaudiana
Centropodia_glauca
Rytidosperma_pallidum
Rytidosperma_semiannulare
Tenaxia_guillarmodiae
Tribolium_hispidum
Danthonia_californica
Chaetobromus_involucratus_subsp._dregeanus
Chionochloa_macra
Phragmites_australis
Hakonechloa_macra
Monachather_paradoxa
Elytrophorus_spicatus
Eriachne_stipacea
Isachne_distichophylla
Micraira_spiciformis
Stipagrostis_hirtigluma
Stipagrostis_uniplumis_var._uniplumis
Sartidia_dewinteri
Sartidia_perrieri
Aristida_ternipes
Aristida_purpurea
Puelia_olyriformis
Pharus_latifolius
Pharus_lappulaceus
Leptaspis_banksii
Leptaspis_zeylanica
Anomochloa_marantoidea
Streptochaeta_spicata
0.01

B

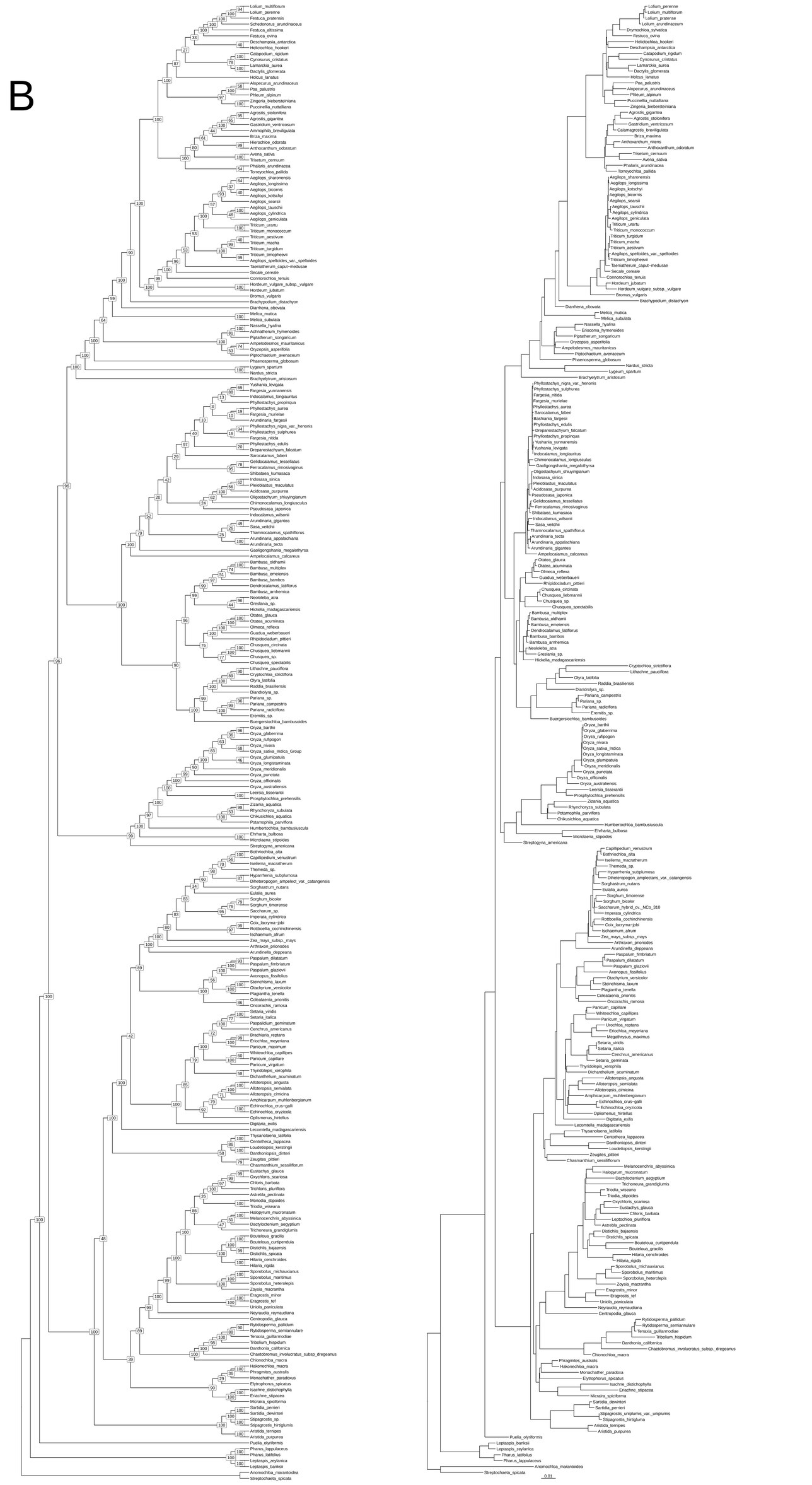
Lolium_multiflorum
Lolium_perenne
Festuca_pratensis
Schedonorus_arundinaceus
Festuca_altissima
Festuca_ovina
Deschampsia_antarctica
Helictochloa_hookeri
Catapodium_rigidum
Cynosurus_cristatus
Lamarckia_aurea
Dactylis_glomerata
Holcus_lanatus
Alopecurus_arundinaceus
Poa_palustris
Phleum_alpinum
Zingeria_biebersteiniana
Puccinellia_nuttalliana
Agrostis_stolonifera
Agrostis_gigantea
Gastridium_ventricosum
Ammophila_breviligulata
Briza_maxima
Hierochloe_odorata
Anthoxanthum_odoratum
Avena_sativa
Trisetum_cernuum
Phalaris_arundinacea
Torreyochloa_pallida
Aegilops_sharonensis
Aegilops_longissima
Aegilops_bicornis
Aegilops_kotschyi
Aegilops_searsii
Aegilops_tauschii
Aegilops_cylindrica
Aegilops_geniculata
Triticum_urartu
Triticum_monococcum
Triticum_aestivum
Triticum_macha
Triticum_turgidum
Triticum_timopheevii
Aegilops_speltoides_var._speltoides
Taeniatherum_caput-medusae
Secale_cereale
Connorochloa_tenuis
Hordeum_vulgare_subsp._vulgare
Hordeum_jubatum
Bromus_vulgaris
Brachypodium_distachyon
Diarrhena_obovata
Melica_mutica
Melica_subulata
Nassella_hyalina
Achnatherum_hymenoides
Piptatherum_songaricum
Ampelodesmos_mauritanicus
Oryzopsis_asperifolia
Piptochaetium_avenaceum
Phaenosperma_globosum
Lygeum_spartum
Nardus_stricta
Brachyelytrum_aristosum
Yushania_levigata
Fargesia_yunnanensis
Indocalamus_longiauritus
Phyllostachys_propinqua
Phyllostachys_aurea
Fargesia_murielae
Arundinaria_fargesii
Phyllostachys_nigra_var._henonis
Phyllostachys_sulphurea
Fargesia_nitida
Phyllostachys_edulis
Drepanostachyum_falcatum
Sarocalamus_faberi
Geldocalamus_tessellatus
Ferrocalamus_rimosivaginus
Shibataea_kumasaca
Indosasa_sinica
Pleioblastus_maculatus
Acidosasa_purpurea
Oligostachyum_shiuyinganum
Chimonocalamus_longiusculus
Pseudosasa_japonica
Indocalamus_wilsonii
Arundinaria_gigantea
Sasa_veitchii
Thamnocalamus_spathiflorus
Arundinaria_appalachiana
Arundinaria_tecta
Gaoligongshania_megalothyrsa
Ampelocalamus_calcareus
Bambusa_oldhamii
Bambusa_multiplex
Bambusa_emeiensis
Bambusa_bambos
Dendrocalamus_latiflorus
Bambusa_arnhemica
Neololeba_atra
Greslania_sp.
Hickelia_madagascariensis
Otatea_glauca
Otatea_acuminata
Olmeca_reflexa
Guadua_weberbaueri
Rhipidocladum_pittieri
Chusquea_circinata
Chusquea_liebmannii
Chusquea_sp.
Chusquea_spectabilis
Lithachne_pauciflora
Cryptochloa_strictiflora
Olyra_latifolia
Raddia_brasiliensis
Diandrolyra_sp.
Pariana_sp.
Pariana_campestris
Pariana_radiciflora
Eremitis_sp.
Buergersiochloa_bambusoides
Oryza_barthii
Oryza_glaberrima
Oryza_rufipogon
Oryza_nivara
Oryza_sativa_Indica_Group
Oryza_glumipatula
Oryza_longistaminata
Oryza_meridionalis
Oryza_punctata
Oryza_officinalis
Oryza_australiensis
Leersia_tisserantii
Prosphytochloa_prehensilis
Zizania_aquatica
Rhynchoryza_subulata
Chikusichloa_aquatica
Potamophila_parviflora
Humbertochloa_bambusiuscula
Ehrharta_bulbosa
Microlaena_stipoides
Streptogyna_americana
Bothriochloa_alta
Capillipedium_venustrum
Iseilema_macratherum
Themeda_sp.
Hyparrhenia_subplumosa
Diheteropogon_amplectans_var._catangensis
Sorghastrum_nutans
Eulalia_aurea
Sorghum_bicolor
Sorghum_timorense
Saccharum_sp.
Imperata_cylindrica
Coix_lacryma-jobi
Rottboellia_cochinchinensis
Ischaemum_afrum
Zea_mays_subsp._mays
Arthraxon_prionodes
Arundinella_deppeana
Paspalum_dilatatum
Paspalum_fimbriatum
Paspalum_glaziovii
Axonopus_fissifolius
Steinchisma_laxum
Otachyrium_versicolor
Plagiantha_tenella
Coleataenia_prionitis
Oncorachis_ramosa
Setaria_viridis
Setaria_italica
Paspalidium_geminatum
Cenchrus_americanus
Brachiaria_reptans
Eriochloa_meyeriana
Panicum_maximum
Whiteochloa_capillipes
Panicum_capillare
Panicum_virgatum
Thyridolepis_xerophila
Dichanthelium_acuminatum
Alloteropsis_angusta
Alloteropsis_semialata
Alloteropsis_cimicina
Amphicarpum_muhlenbergianum
Echinochloa_crus-galli
Echinochloa_oryzicola
Oplismenus_hirtellus
Digitaria_exilis
Lecomtella_madagascariensis
Thysanolaena_latifolia
Centotheca_lappacea
Loudetopsis_kerstingii
Danthoniopsis_dinteri
Zeugites_pittieri
Chasmanthium_sessiliflorum
Eustachys_glauca
Oxychloris_scariosa
Chloris_barbata
Trichloris_pluriflora
Astrebla_pectinata
Monodia_stipoides
Triodia_wiseana
Halopyrum_mucronatum
Melanocenchris_abyssinica
Dactyloctenium_aegyptium
Trichoneura_grandiglumis
Bouteloua_gracilis
Bouteloua_curtipendula
Distichlis_bajaensis
Distichlis_spicata
Hilaria_cenchroides
Hilaria_rigida
Sporobolus_michauxianus
Sporobolus_maritimus
Sporobolus_heterolepis
Zoysia_macrantha
Eragrostis_minor
Eragrostis_tef
Uniola_paniculata
Neyraudia_reynaudiana
Centropodia_glauca
Rytidosperma_pallidum
Rytidosperma_semiannulare
Tenaxia_guillarmodiae
Tribolium_hispidum
Danthonia_californica
Chaetobromus_involucratus_subsp._dregeanus
Chionochloa_macra
Hakonechloa_macra
Phragmites_australis
Monachather_paradoxa
Elytrophorus_spicatus
Isachne_distichophylla
Eriachne_stipacea
Micraira_spiciformis
Sartidia_perrieri
Sartidia_dewinteri
Stipagrostis_sp.
Stipagrostis_hirtigluma
Aristida_ternipes
Aristida_purpurea
Puelia_olyriformis
Pharus_lappulaceus
Pharus_latifolius
Leptaspis_zeylanica
Leptaspis_banksii
Anomochloa_marantoidea
Streptochaeta_spicata
0.01

C

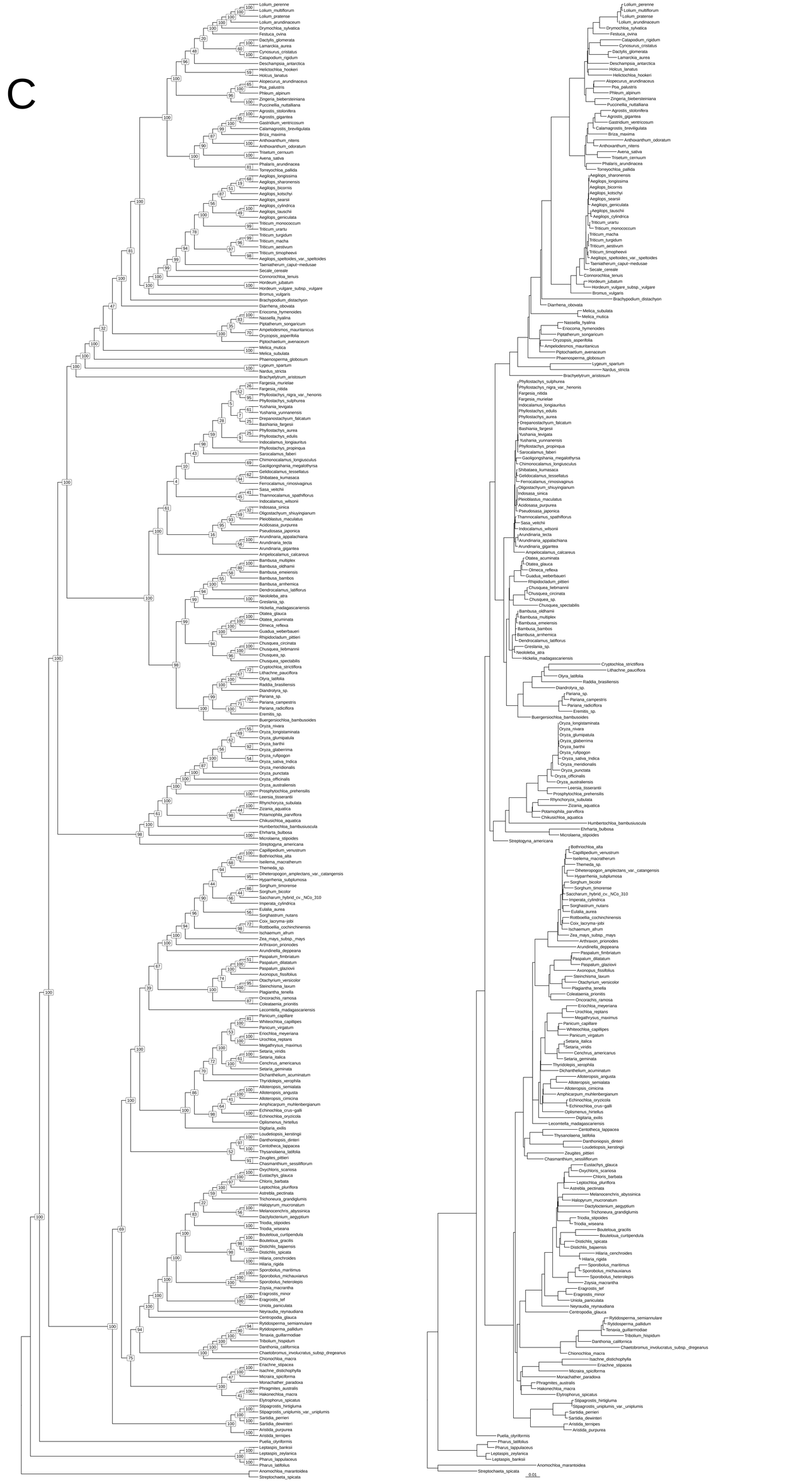
Lolium_perenne
Lolium_multiflorum
Lolium_pratense
Lolium_arundinaceum
Drymochloa_sylvatica
Festuca_ovina
Catapodium_rigidum
Cynosurus_cristatus
Dactylis_glomerata
Lamarckia_aurea
Deschampsia_antarctica
Holcus_lanatus
Helictochloa_hookeri
Alopecurus_arundinaceus
Poa_palustris
Phleum_alpinum
Zingeria_biebersteiniana
Puccinellia_nuttalliana
Agrostis_stolonifera
Agrostis_gigantea
Gastridium_ventricosum
Calamagrostis_breviligulata
Briza_maxima
Anthoxanthum_nitens
Anthoxanthum_odoratum
Trisetum_cernuum
Avena_sativa
Phalaris_arundinacea
Torreyochloa_pallida
Aegilops_longissima
Aegilops_sharonensis
Aegilops_bicornis
Aegilops_kotschyi
Aegilops_searsii
Aegilops_cylindrica
Aegilops_tauschii
Aegilops_geniculata
Triticum_monococcum
Triticum_urartu
Triticum_turgidum
Triticum_macha
Triticum_aestivum
Triticum_timopheevii
Aegilops_speltoides_var_speltoides
Taeniatherum_caput-medusae
Secale_cereale
Connorochloa_tenuis
Hordeum_jubatum
Hordeum_vulgare_subsp._vulgare
Bromus_vulgaris
Brachypodium_distachyon
Diarrhena_obovata
Melica_subulata
Melica_mutica
Nassella_hyalina
Eriocoma_hymenoides
Piptatherum_songaricum
Oryzopsis_asperifolia
Ampelodesmos_mauritanicus
Piptochaetium_avenaceum
Phaenosperma_globosum
Lygeum_spartum
Nardus_stricta
Brachyelytrum_aristosum
Phyllostachys_sulphurea
Phyllostachys_nigra_var._henonis
Fargesia_nitida
Fargesia_murielae
Indocalamus_longiauritus
Phyllostachys_edulis
Phyllostachys_aurea
Drepanostachyum_falcatum
Bashania_fargesii
Yushania_levigata
Yushania_yunnanensis
Phyllostachys_propinqua
Sarocalamus_faberi
Gaoligongshania_megalothyrsa
Chimonocalamus_longiusculus
Shibataea_kumasaca
Gelidocalamus_tessellatus
Ferrocalamus_rimosivaginus
Oligostachyum_shiuyingianum
Indosasa_sinica
Pleioblastus_maculatus
Acidosasa_purpurea
Pseudosasa_japonica
Thamnocalamus_spathiflorus
Sasa_vetchii
Indocalamus_wilsonii
Arundinaria_tecta
Arundinaria_appalachiana
Arundinaria_gigantea
Ampelocalamus_calcareus
Otatea_acuminata
Otatea_glauca
Olmeca_reflexa
Guadua_weberbaueri
Rhipidocladum_pittieri
Chusquea_liebmannii
Chusquea_circinata
Chusquea_sp.
Chusquea_spectabilis
Bambusa_oldhamii
Bambusa_multiplex
Bambusa_emeiensis
Bambusa_bambos
Bambusa_arnhemica
Dendrocalamus_latiflorus
Greslania_sp.
Neololeba_atra
Hickelia_madagascariensis
Cryptochloa_strictiflora
Lithachne_pauciflora
Olyra_latifolia
Raddia_brasiliensis
Diandrolyra_sp.
Pariana_sp.
Pariana_campestris
Pariana_radiciflora
Eremitis_sp.
Buergersiochloa_bambusoides
Oryza_longistaminata
Oryza_nivara
Oryza_glumipatula
Oryza_glaberrima
Oryza_barthii
Oryza_rufipogon
Oryza_sativa_Indica
Oryza_meridionalis
Oryza_punctata
Oryza_officinalis
Oryza_australiensis
Prosphytochloa_prehensilis
Leersia_tisserantii
Rhynchoryza_subulata
Zizania_aquatica
Potamophila_parviflora
Chikusichloa_aquatica
Humbertochloa_bambusiuscula
Ehrharta_bulbosa
Microlaena_stipoides
Streptogyna_americana
Capillipedium_venustrum
Bothriochloa_alta
Iselema_macratherum
Themeda_sp.
Diheteropogon_amplectans_var._catangensis
Hyparrhenia_subplumosa
Sorghum_bicolor
Sorghum_timorense
Saccharum_hybrid_cv._NCo_310
Imperata_cylindrica
Sorghastrum_nutans
Eulalia_aurea
Rottboellia_cochinchinensis
Coix_lacryma-jobi
Ischaemum_afrum
Zea_mays_subsp._mays
Arthraxon_prionodes
Arundinella_deppeana
Paspalum_fimbriatum
Paspalum_dilatatum
Paspalum_glaziovii
Axonopus_fissifolius
Steinchisma_laxum
Otachyrium_versicolor
Plagiantha_tenella
Coleataenia_prionitis
Oncorachis_ramosa
Eriochloa_meyeriana
Urochloa_reptans
Megathrysus_maximus
Panicum_capillare
Whiteochloa_capillipes
Panicum_virgatum
Setaria_italica
Setaria_viridis
Cenchrus_americanus
Setaria_geminata
Thyridolepis_xerophila
Dichanthelium_acuminatum
Alloteropsis_angusta
Alloteropsis_semialata
Alloteropsis_cimicina
Amphicarpum_muhlenbergianum
Echinochloa_oryzicola
Echinochloa_crus-galli
Oplismenus_hirtellus
Digitaria_exilis
Lecomtella_madagascariensis
Centotheca_lappacea
Thysanolaena_latifolia
Danthoniopsis_dinteri
Loudetopsis_kerstingii
Zeugites_pittieri
Chasmanthium_sessiliflorum
Eustachys_glauca
Oxychloris_scariosa
Chloris_barbata
Leptochloa_pluriflora
Astrebla_pectinata
Melanocenchris_abyssinica
Halopyrum_mucronatum
Dactyloctenium_aegyptium
Trichoneura_grandiglumis
Triodia_stipoides
Triodia_wiseana
Bouteloua_gracilis
Bouteloua_curtipendula
Distichlis_spicata
Distichlis_bajaensis
Hilaria_cenchroides
Hilaria_rigida
Sporobolus_maritimus
Sporobolus_michauxianus
Sporobolus_heterolepis
Zoysia_macrantha
Eragrostis_minor
Eragrostis_tef
Uniola_paniculata
Neyraudia_reynaudiana
Centropodia_glauca
Rytidosperma_semiannulare
Rytidosperma_pallidum
Tenaxia_guillarmodiae
Tribolium_hispidum
Danthonia_californica
Chaetobromus_involucratus_subsp._dregeanus
Chionochloa_macra
Eriachne_stipacea
Isachne_distichophylla
Micraira_spiciformis
Monachather_paradoxa
Phragmites_australis
Hakonechloa_macra
Elytrophorus_spicatus
Stipagrostis_hirtigluma
Stipagrostis_uniplumis_var._uniplumis
Sartidia_perrieri
Sartidia_dewinteri
Aristida_purpurea
Aristida_ternipes
Puelia_olyriformis
Pharus_latifolius
Pharus_lappulaceus
Leptaspis_zeylanica
Leptaspis_banksii
Anomochloa_marantoidea
Streptochaeta_spicata
0.01

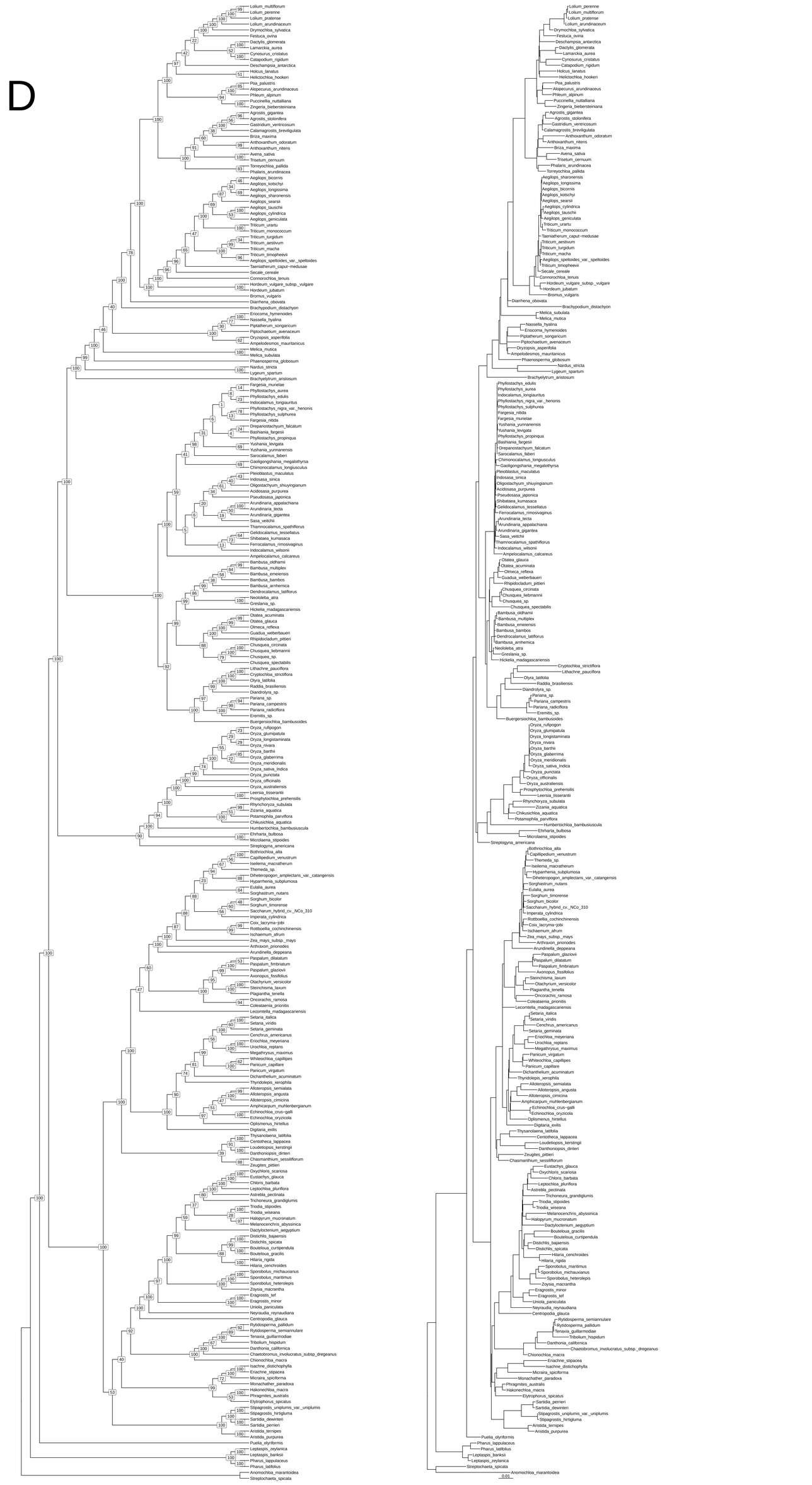
D
Lolium_multiflorum
Lolium_perenne
Lolium_pratense
Lolium_arundinaceum
Drymochloa_sylvatica
Festuca_ovina
Dactylis_glomerata
Lamarckia_aurea
Cynosurus_cristatus
Catapodium_rigidum
Deschampsia_antarctica
Holcus_lanatus
Helictochloa_hookeri
Poa_palustris
Alopecurus_arundinaceus
Phleum_alpinum
Puccinellia_nuttalliana
Zingeria_biebersteiniana
Agrostis_gigantea
Agrostis_stolonifera
Gastridium_ventricosum
Calamagrostis_breviligulata
Briza_maxima
Anthoxanthum_odoratum
Anthoxanthum_nitens
Avena_sativa
Trisetum_cernuum
Torreyochloa_pallida
Phalaris_arundinacea
Aegilops_bicornis
Aegilops_kotschyi
Aegilops_longissima
Aegilops_sharonensis
Aegilops_searsii
Aegilops_tauschii
Aegilops_cylindrica
Aegilops_geniculata
Triticum_urartu
Triticum_monococcum
Triticum_turgidum
Triticum_aestivum
Triticum_macha
Triticum_timopheevii
Aegilops_speltoides_var._speltoides
Taeniatherum_caput-medusae
Secale_cereale
Connorochloa_tenuis
Hordeum_vulgare_subsp._vulgare
Hordeum_jubatum
Bromus_vulgaris
Diarrhena_obovata
Brachypodium_distachyon
Eriocoma_hymenoides
Nassella_hyalina
Piptatherum_songaricum
Piptochaetium_avenaceum
Oryzopsis_asperifolia
Ampeldesmos_mauritanicus
Melica_mutica
Melica_subulata
Phaenosperma_globosum
Nardus_stricta
Lygeum_spartum
Brachyelytrum_aristosum
Fargesia_murielae
Phyllostachys_aurea
Phyllostachys_edulis
Indocalamus_longiauritus
Phyllostachys_nigra_var._henonis
Phyllostachys_sulphurea
Fargesia_nitida
Dregeanostachyum_falcatum
Bashania_fargesii
Phyllostachys_propinqua
Yushania_levigata
Yushania_yunnanensis
Sarocalamus_faberi
Gaoligongshania_megalothyrsa
Chimonocalamus_longiusculus
Plioblastus_maculatus
Indosasa_sinica
Oligostachyum_shiuyingianum
Acidosasa_purpurea
Pseudosasa_japonica
Arundinaria_appalachiana
Arundinaria_tecta
Arundinaria_gigantea
Sasa_veitchii
Thamnocalamus_spathiflorus
Geldocalamus_tessellatus
Shibataea_kumasaca
Ferrocalamus_rimosivaginus
Indocalamus_wilsonii
Ampelocalamus_calcareus
Bambusa_oldhamii
Bambusa_multiplex
Bambusa_emeiensis
Bambusa_bambos
Bambusa_arnhemica
Dendrocalamus_latiflorus
Neololeba_atra
Greslania_sp.
Hickelia_madagascariensis
Otatea_acuminata
Otatea_glauca
Olmeca_reflexa
Guadua_weberbaueri
Rhipidocladum_pittieri
Chusquea_circinata
Chusquea_liebmannii
Chusquea_sp.
Chusquea_spectabilis
Lithachne_pauciflora
Cryptochloa_strictiflora
Olyra_latifolia
Raddia_brasiliensis
Diandrolyra_sp.
Pariana_sp.
Pariana_campestris
Pariana_radiciflora
Eremitis_sp.
Buergersiochloa_bambusoides
Oryza_rufipogon
Oryza_glumipatula
Oryza_longistaminata
Oryza_nivara
Oryza_barthii
Oryza_glaberrima
Oryza_meridionalis
Oryza_sativa_Indica
Oryza_punctata
Oryza_officinalis
Oryza_australiensis
Leersia_tisserantii
Prosphytochloa_prehensilis
Rhynchoryza_subulata
Zizania_aquatica
Chikuschloa_aquatica
Potamophila_parviflora
Humbertochloa_bambusiuscula
Ehrharta_bulbosa
Microlaena_stipoides
Streptogyna_americana
Bothriochloa_alta
Capillipedium_venustrum
Themeda_sp.
Iseilema_macratherum
Hyparrhenia_subplumosa
Dihetеropogon_amplectans_var._catangensis
Sorghastrum_nutans
Eulalia_aurea
Sorghum_timorense
Sorghum_bicolor
Saccharum_hybrid_cv._NCo_310
Imperata_cylindrica
Coix_lacryma-jobi
Rottboellia_cochinchinensis
Ischaemum_afrum
Zea_mays_subsp._mays
Arthraxon_prionodes
Arundinella_deppeana
Paspalum_dilatatum
Paspalum_fimbriatum
Paspalum_glazovii
Axonopus_fissifolius
Steinchisma_laxum
Otachyrium_versicolor
Plagiantha_tenella
Oncorachis_ramosa
Coleataenia_prionitis
Lecomtella_madagascariensis
Setaria_italica
Setaria_viridis
Setaria_geminata
Cenchrus_americanus
Eriochloa_meyeriana
Urochloa_reptans
Megathrysus_maximus
Whiteochloa_capillipes
Panicum_capillare
Panicum_virgatum
Dichanthelium_acuminatum
Thyridolepis_xerophila
Alloteropsis_semialata
Alloteropsis_angusta
Alloteropsis_cimicina
Amphicarpum_muhlenbergianum
Echinochloa_crus-galli
Echinochloa_oryzicola
Oplismenus_hirtellus
Digitaria_exilis
Thysanolaena_latifolia
Centotheca_lappacea
Loudetiopsis_kerstingii
Danthoniopsis_dinteri
Zeugites_pittieri
Chasmanthium_sessiliflorum
Eustachys_glauca
Oxychloris_scariosa
Chloris_barbata
Leptochloa_pluriflora
Astrebla_pectinata
Trichoneura_grandiglumis
Triodia_stipoides
Triodia_wiseana
Halopyrum_mucronatum
Melanocenchris_abyssinica
Dactyloctenium_aegyptium
Distichlis_bajaensis
Distichlis_spicata
Bouteloua_curtipendula
Bouteloua_gracilis
Hilaria_rigida
Hilaria_cenchroides
Sporobolus_michauxianus
Sporobolus_maritimus
Sporobolus_heterolepis
Zoysia_macrantha
Eragrostis_tef
Eragrostis_minor
Uniola_paniculata
Neyraudia_reynaudiana
Centropodia_glauca
Rytidosperma_pallidum
Rytidosperma_semiannulare
Tenaxia_guillarmodiae
Tribolium_hispidum
Danthonia_californica
Chaetobromus_involucratus_subsp._dregeanus
Chionochloa_macra
Isachne_distichophylla
Eriachne_stipacea
Micraira_spiciformа
Monachather_paradoxa
Hakonechloa_macra
Phragmites_australis
Elytrophorus_spicatus
Stipagrostis_uniplumis_var._uniplumis
Stipagrostis_hirtigluma
Sartidia_dewinteri
Sartidia_perrieri
Aristida_ternipes
Aristida_purpurea
Puelia_olyriformis
Leptaspis_zeylanica
Leptaspis_banksii
Pharus_lappulaceus
Pharus_latifolius
Anomochloa_marantoidea
Streptochaeta_spicata
0.01

E

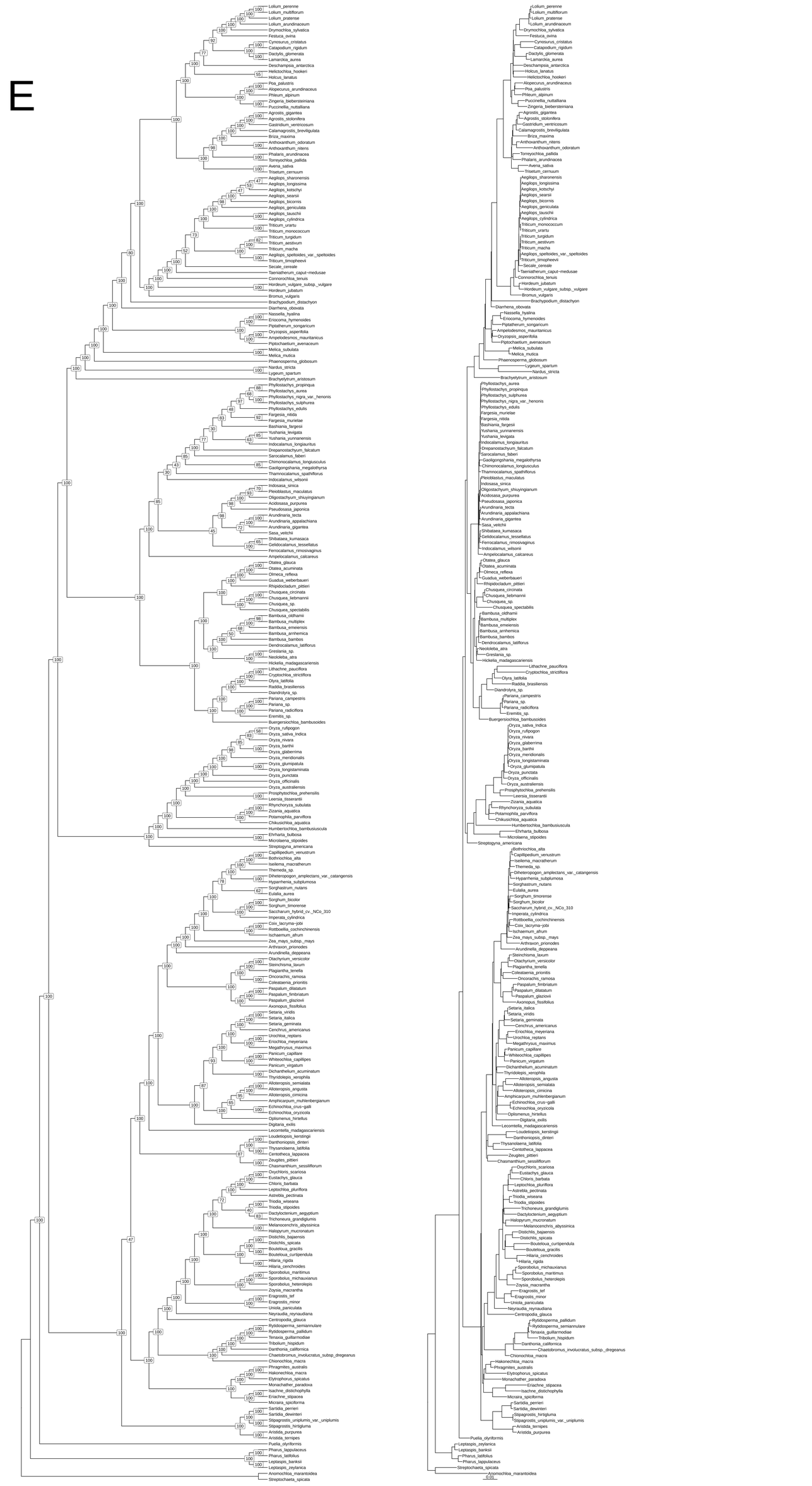
Lolium_perenne
Lolium_multiflorum
Lolium_pratense
Lolium_arundinaceum
Drymochloa_sylvatica
Festuca_ovina
Cynosurus_cristatus
Catapodium_rigidum
Dactylis_glomerata
Lamarckia_aurea
Deschampsia_antarctica
Helictochloa_hookeri
Holcus_lanatus
Poa_palustris
Alopecurus_arundinaceus
Phleum_alpinum
Zingeria_biebersteiniana
Puccinellia_nuttalliana
Agrostis_gigantea
Agrostis_stolonifera
Gastridium_ventricosum
Calamagrostis_brevilig
Briza_maxima
Anthoxanthum_odoratum
Anthoxanthum_nitens
Phalaris_arundinacea
Toreyochloa_pallida
Avena_sativa
Trisetum_cernuum
Aegilops_sharonensis
Aegilops_longissima
Aegilops_kotschyi
Aegilops_searsii
Aegilops_bicornis
Aegilops_geniculata
Aegilops_tauschii
Aegilops_cylindrica
Triticum_urartu
Triticum_monococcum
Triticum_turgidum
Triticum_aestivum
Triticum_macha
Aegilops_speltoides_var._speltoides
Triticum_timopheevii
Secale_cereale
Taeniatherum_caput-medusae
Connorochloa_tenuis
Hordeum_vulgare_subsp._vulgare
Hordeum_jubatum
Bromus_vulgaris
Brachypodium_distachyon
Diarrhena_obovata
Nassella_hyalina
Eriocoma_hymenoides
Piptatherum_songaricum
Oryzopsis_asperifolia
Ampelodesmos_mauritanicus
Piptochaetium_avenaceum
Melica_subulata
Melica_mutica
Phaenosperma_globosum
Nardus_stricta
Lygeum_spartum
Brachyelytrum_aristosum
Phyllostachys_propinqua
Phyllostachys_aurea
Phyllostachys_nigra_var._henonis
Phyllostachys_sulphurea
Phyllostachys_edulis
Fargesia_murielae
Fargesia_nitida
Bashiania_fargesii
Yushania_levigata
Yushania_yunnanensis
Indocalamus_longiauritus
Drepanostachyum_falcatum
Sarocalamus_faberi
Chimonocalamus_longiusculus
Gaoligongshania_megalothyrsa
Thamnocalamus_spathiflorus
Indocalamus_wilsonii
Indosasa_sinica
Pleioblastus_maculatus
Oligostachyum_shiuyinganum
Acidosasa_purpurea
Pseudosasa_japonica
Arundinaria_tecta
Arundinaria_appalachiana
Arundinaria_gigantea
Sasa_veitchii
Shibataea_kumasaca
Gelidocalamus_tessellatus
Ferrocalamus_rimosivaginus
Ampelocalamus_calcareus
Otatea_glauca
Otatea_acuminata
Olmeca_reflexa
Guadua_weberbaueri
Rhipidocladum_pittieri
Chusquea_circinata
Chusquea_liebmannii
Chusquea_sp.
Chusquea_spectabilis
Bambusa_oldhamii
Bambusa_multiplex
Bambusa_emeiensis
Bambusa_arnhemica
Bambusa_bambos
Dendrocalamus_latiflorus
Greslania_sp.
Neololeba_atra
Hickelia_madagascariensis
Lithachne_pauciflora
Cryptochloa_strictiflora
Olyra_latifolia
Raddia_brasiliensis
Diandrolyra_sp.
Pariana_campestris
Pariana_sp.
Pariana_radiciflora
Eremitis_sp.
Buergersiochloa_bambusoides
Oryza_rufipogon
Oryza_sativa_Indica
Oryza_nivara
Oryza_barthii
Oryza_glaberrima
Oryza_meridionalis
Oryza_glumipatula
Oryza_longistaminata
Oryza_punctata
Oryza_officinalis
Oryza_australiensis
Prosphytochloa_prehensilis
Leersia_tisserantii
Rhynchoryza_subulata
Zizania_aquatica
Potamophila_parviflora
Chikusichloa_aquatica
Humbertochloa_bambusiuscula
Ehrharta_bulbosa
Microlaena_stipoides
Streptogyna_americana
Capillipedium_venustrum
Bothriochloa_alta
Iseilema_macratherum
Themeda_sp.
Diheteropogon_amplectans_var._catangensis
Hyparrhenia_subplumosa
Sorghastrum_nutans
Eulalia_aurea
Sorghum_bicolor
Sorghum_timorense
Saccharum_hybrid_cv._NCo_310
Imperata_cylindrica
Coix_lacryma-jobi
Rottboellia_cochinchinensis
Ischaemum_afrum
Zea_mays_subsp._mays
Arthraxon_prionodes
Arundinella_deppeana
Otachyrium_versicolor
Steinchisma_laxum
Plagiantha_tenella
Oncorachis_ramosa
Coleataenia_prionitis
Paspalum_dilatatum
Paspalum_fimbriatum
Paspalum_glazovii
Axonopus_fissifolius
Setaria_viridis
Setaria_italica
Setaria_geminata
Cenchrus_americanus
Urochloa_reptans
Eriochloa_meyeriana
Megathrysus_maximus
Panicum_capillare
Whiteochloa_capillipes
Panicum_virgatum
Dichanthelium_acuminatum
Thyridolepis_xerophila
Alloteropsis_semialata
Alloteropsis_angusta
Alloteropsis_cimicina
Amphicarpum_muhlenbergianum
Echinochloa_crus-galli
Echinochloa_oryzicola
Oplismenus_hirtellus
Digitaria_exilis
Lecomtella_madagascariensis
Loudetopsis_kerstingii
Danthoniopsis_dinteri
Thysanolaena_latifolia
Centotheca_lappacea
Zeugites_pittieri
Chasmanthium_sessiliflorum
Oxychloris_scariosa
Eustachys_glauca
Chloris_barbata
Leptochloa_pluriflora
Astrebla_pectinata
Triodia_wiseana
Triodia_stipoides
Dactyloctenium_aegyptium
Trichoneura_grandiglumis
Melanocenchris_abyssinica
Halopyrum_mucronatum
Distichlis_bajaensis
Distichlis_spicata
Bouteloua_curtipendula
Bouteloua_gracilis
Hilaria_rigida
Hilaria_cenchroides
Sporobolus_maritimus
Sporobolus_michauxianus
Sporobolus_heterolepis
Zoysia_macrantha
Eragrostis_tef
Eragrostis_minor
Uniola_paniculata
Neyraudia_reynaudiana
Centropodia_glauca
Rytidosperma_semiannulare
Rytidosperma_pallidum
Tenaxia_guillarmodiae
Tribolium_hispidum
Danthonia_californica
Chaetobromus_involucratus_subsp._dregeanus
Chionochloa_macra
Phragmites_australis
Hakonechloa_macra
Elytrophorus_spicatus
Monachather_paradoxa
Isachne_distichophylla
Eriachne_stipacea
Micraira_spiciformis
Sartidia_perrieri
Sartidia_dewinteri
Stipagrostis_uniplumis_var._uniplumis
Stipagrostis_hirtigluma
Aristida_purpurea
Aristida_ternipes
Puelia_olyriformis
Pharus_lappulaceus
Pharus_latifolius
Leptaspis_banksii
Leptaspis_zeylanica
Anomochloa_marantoidea
Streptochaeta_spicata
0.01

F

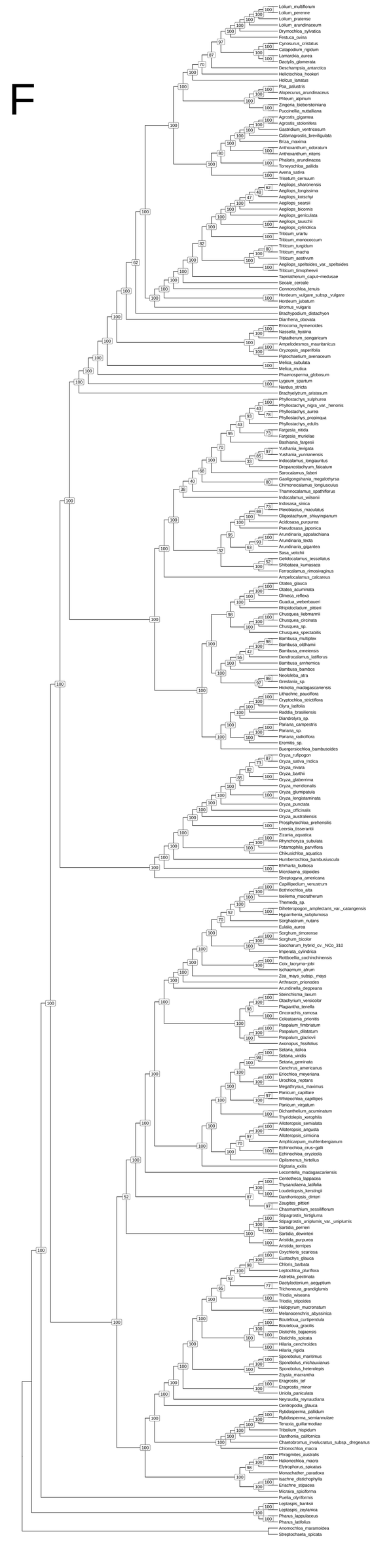
Lolium_multiflorum
Lolium_perenne
Lolium_pratense
Lolium_arundinaceum
Drymochloa_sylvatica
Festuca_ovina
Cynosurus_cristatus
Catapodium_rigidum
Lamarckia_aurea
Dactylis_glomerata
Deschampsia_antarctica
Helictochloa_hookeri
Holcus_lanatus
Poa_palustris
Alopecurus_arundinaceus
Phleum_alpinum
Zingeria_biebersteiniana
Puccinellia_nuttalliana
Agrostis_gigantea
Agrostis_stolonifera
Gastridium_ventricosum
Calamagrostis_breviligulata
Briza_maxima
Anthoxanthum_odoratum
Anthoxanthum_nitens
Phalaris_arundinacea
Toreyochloa_pallida
Avena_sativa
Trisetum_cernuum
Aegilops_sharonensis
Aegilops_longissima
Aegilops_kotschyi
Aegilops_searsii
Aegilops_bicornis
Aegilops_geniculata
Aegilops_tauschii
Aegilops_cylindrica
Triticum_urartu
Triticum_monococcum
Triticum_turgidum
Triticum_macha
Triticum_aestivum
Aegilops_speltoides_var._speltoides
Triticum_timopheevii
Taeniatherum_caput-medusae
Secale_cereale
Connorochloa_tenuis
Hordeum_vulgare_subsp._vulgare
Hordeum_jubatum
Bromus_vulgaris
Brachypodium_distachyon
Diarrhena_obovata
Eriocoma_hymenoides
Nassella_hyalina
Piptatherum_songaricum
Ampelodesmos_mauritanicus
Oryzopsis_asperifolia
Piptochaetium_avenaceum
Melica_subulata
Melica_mutica
Phaenosperma_globosum
Lygeum_spartum
Nardus_stricta
Brachyelytrum_aristosum
Phyllostachys_sulphurea
Phyllostachys_nigra_var._henonis
Phyllostachys_aurea
Phyllostachys_propinqua
Phyllostachys_edulis
Fargesia_nitida
Fargesia_murielae
Bashania_fargesii
Yushania_levigata
Yushania_yunnanensis
Indocalamus_longiauritus
Drepanostachyum_falcatum
Sarocalamus_faberi
Gaoligongshania_megalothyrsa
Chimonocalamus_longiusculus
Thamnocalamus_spathiflorus
Indocalamus_wilsonii
Indosasa_sinica
Pleioblastus_maculatus
Oligostachyum_shiuyingianum
Acidosasa_purpurea
Pseudosasa_japonica
Arundinaria_appalachiana
Arundinaria_tecta
Arundinaria_gigantea
Sasa_veitchii
Gelidocalamus_tessellatus
Shibataea_kumasaca
Ferrocalamus_rimosivaginus
Ampelocalamus_calcareus
Otatea_glauca
Otatea_acuminata
Olmeca_reflexa
Guadua_weberbaueri
Rhipidocladum_pittieri
Chusquea_liebmannii
Chusquea_circinata
Chusquea_sp.
Chusquea_spectabilis
Bambusa_multiplex
Bambusa_oldhamii
Bambusa_emeiensis
Dendrocalamus_latiflorus
Bambusa_arnhemica
Bambusa_bambos
Neololeba_atra
Greslania_sp.
Hickelia_madagascariensis
Lithachne_pauciflora
Cryptochloa_strictiflora
Olyra_latifolia
Raddia_brasiliensis
Diandrolyra_sp.
Pariana_campestris
Pariana_sp.
Pariana_radiciflora
Eremitis_sp.
Buergersiochloa_bambusoides
Oryza_rufipogon
Oryza_sativa_Indica
Oryza_nivara
Oryza_barthii
Oryza_glaberrima
Oryza_meridionalis
Oryza_glumipatula
Oryza_longistaminata
Oryza_punctata
Oryza_officinalis
Oryza_australiensis
Prosphytochloa_prehensilis
Leersia_tisserantii
Zizania_aquatica
Rhynchoryza_subulata
Potamophila_parviflora
Chikusichloa_aquatica
Humbertochloa_bambusiuscula
Ehrharta_bulbosa
Microlaena_stipoides
Streptogyna_americana
Capillipedium_venustrum
Bothriochloa_alta
Iseilema_macratherum
Themeda_sp.
Diheteropogon_amplectans_var._catangensis
Hyparrhenia_subplumosa
Sorghastrum_nutans
Eulalia_aurea
Sorghum_timorense
Sorghum_bicolor
Saccharum_hybrid_cv._NCo_310
Imperata_cylindrica
Rottboellia_cochinchinensis
Coix_lacryma-jobi
Ischaemum_afrum
Zea_mays_subsp._mays
Arthraxon_prionodes
Arundinella_deppeana
Otachyrium_versicolor
Steinchisma_laxum
Plagiantha_tenella
Coleataenia_prionitis
Oncorachis_ramosa
Paspalum_fimbriatum
Paspalum_dilatatum
Paspalum_glazovii
Axonopus_fissifolius
Setaria_italica
Setaria_viridis
Setaria_germinata
Cenchrus_americanus
Eriochloa_meyeriana
Urochloa_reptans
Megathrysus_maximus
Panicum_capillare
Whiteochloa_capillipes
Panicum_virgatum
Dichanthelium_acuminatum
Thyridolepis_xerophila
Alloteropsis_semialata
Alloteropsis_angusta
Alloteropsis_cimicina
Amphicarpum_muhlenbergianum
Echinochloa_crus-galli
Echinochloa_oryzicola
Oplismenus_hirtellus
Digitaria_exilis
Lecomtella_madagascariensis
Centotheca_lappacea
Thysanolaena_latifolia
Loudetopsis_kerstingii
Danthoniopsis_dinteri
Zeugites_pittieri
Chasmanthium_sessiliflorum
Stipagrostis_hirtigluma
Stipagrostis_uniplumis_var._uniplumis
Sartidia_perrieri
Sartidia_dewinteri
Aristida_purpurea
Aristida_ternipes
Oxychloris_scariosa
Eustachys_glauca
Chloris_barbata
Leptochloa_pluriflora
Astrebla_pectinata
Dactyloctenium_aegyptium
Trichoneura_grandiglumis
Triodia_wiseana
Triodia_stipoides
Halopyrum_mucronatum
Melanocenchris_abyssinica
Bouteloua_curtipendula
Bouteloua_gracilis
Distichlis_bajaensis
Distichlis_spicata
Hilaria_cenchroides
Hilaria_rigida
Sporobolus_maritimus
Sporobolus_michauxianus
Sporobolus_heterolepis
Zoysia_macrantha
Eragrostis_tef
Eragrostis_minor
Uniola_paniculata
Neyraudia_reynaudiana
Centropodia_glauca
Rytidosperma_pallidum
Rytidosperma_semiannulare
Tenaxia_guillarmodiae
Tribolium_hispidum
Danthonia_californica
Chaetobromus_involucratus_subsp._dregeanus
Chionochloa_macra
Phragmites_australis
Hakonechloa_macra
Elytrophorus_spicatus
Monachather_paradoxa
Isachne_distichophylla
Eriachne_stipacea
Micraira_spiciformis
Puella_olyriformis
Leptaspis_banksii
Leptaspis_zeylanica
Pharus_lappulaceus
Pharus_latifolius
Anomochloa_marantoidea
Streptochaeta_spicata
0.01

G

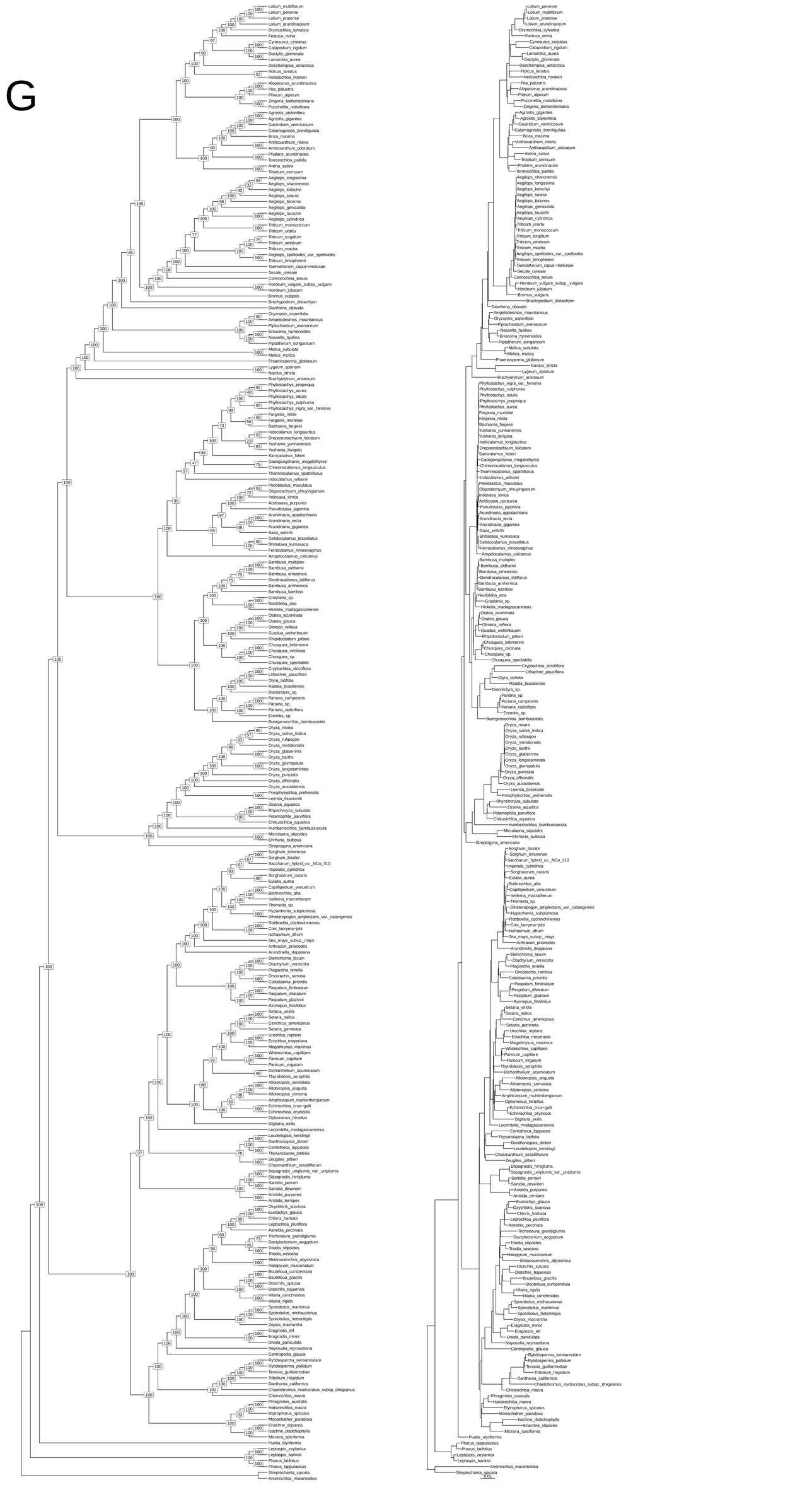
Lolium_multiflorum
Lolium_perenne
Lolium_pratense
Lolium_arundinaceum
Drymochloa_sylvatica
Festuca_ovina
Cynosurus_cristatus
Catapodium_rigidum
Dactylis_glomerata
Lamarckia_aurea
Deschampsia_antarctica
Holcus_lanatus
Helictochloa_hookeri
Alopecurus_arundinaceus
Poa_palustris
Phleum_alpinum
Zingeria_biebersteiniana
Puccinellia_nuttalliana
Agrostis_stolonifera
Agrostis_gigantea
Gastridium_ventricosum
Calamagrostis_brevilgulata
Briza_maxima
Anthoxanthum_nitens
Anthoxanthum_odoratum
Phalaris_arundinacea
Torreyochloa_pallida
Avena_sativa
Trisetum_cernuum
Aegilops_longissima
Aegilops_sharonensis
Aegilops_kotschyi
Aegilops_searsii
Aegilops_bicornis
Aegilops_geniculata
Aegilops_tauschii
Aegilops_cylindrica
Triticum_monococcum
Triticum_urartu
Triticum_turgidum
Triticum_aestivum
Triticum_macha
Aegilops_speltoides_var._speltoides
Triticum_timopheevii
Taeniatherum_caput−medusae
Secale_cereale
Connorochloa_tenuis
Hordeum_vulgare_subsp._vulgare
Hordeum_jubatum
Bromus_vulgaris
Brachypodium_distachyon
Diarrhena_obovata
Oryzopsis_asperifolia
Ampelodesmos_mauritanicus
Piptochaetium_avenaceum
Eriocoma_hymenoides
Nassella_hyalina
Piptatherum_songaricum
Melica_subulata
Melica_mutica
Phaenosperma_globosum
Lygeum_spartum
Nardus_stricta
Brachyelytrum_aristosum
Phyllostachys_propinqua
Phyllostachys_aurea
Phyllostachys_edulis
Phyllostachys_sulphurea
Phyllostachys_nigra_var._henonis
Fargesia_nitida
Fargesia_murielae
Bashania_fargesii
Indocalamus_longiauritus
Drepanostachyum_falcatum
Yushania_yunnanensis
Yushania_levigata
Sarocalamus_faberi
Gaoligongshania_megalothyrsa
Chimonocalamus_longiusculus
Thamnocalamus_spathiflorus
Indocalamus_wilsonii
Pleioblastus_maculatus
Oligostachyum_shiuyingianum
Indosasa_sinica
Acidosasa_purpurea
Pseudosasa_japonica
Arundinaria_appalachiana
Arundinaria_tecta
Arundinaria_gigantea
Sasa_veitchii
Geldocalamus_tessellatus
Shibataea_kumasaca
Ferrocalamus_rimosivaginus
Ampelocalamus_calcareus
Bambusa_multiplex
Bambusa_oldhamii
Bambusa_emeiensis
Dendrocalamus_latiflorus
Bambusa_arnhemica
Bambusa_bambos
Greslania_sp.
Neololeba_atra
Hickelia_madagascariensis
Otatea_acuminata
Otatea_glauca
Olmeca_reflexa
Guadua_weberbaueri
Rhipidocladum_pittieri
Chusquea_liebmannii
Chusquea_circinata
Chusquea_sp.
Chusquea_spectabilis
Cryptochloa_strictiflora
Lithachne_pauciflora
Olyra_latifolia
Raddia_brasiliensis
Diandrolyra_sp.
Pariana_campestris
Pariana_sp.
Pariana_radiciflora
Eremitis_sp.
Buergersiochloa_bambusoides
Oryza_nivara
Oryza_sativa_Indica
Oryza_rufipogon
Oryza_meridionalis
Oryza_glaberrima
Oryza_barthii
Oryza_glumipatula
Oryza_longistaminata
Oryza_punctata
Oryza_officinalis
Oryza_australiensis
Prosphytochloa_prehensilis
Leersia_tisserantii
Zizania_aquatica
Rhynchoryza_subulata
Potamophila_parviflora
Chikusichloa_aquatica
Humbertochloa_bambusiuscula
Microlaena_stipoides
Ehrharta_bulbosa
Streptogyna_americana
Sorghum_bicolor
Sorghum_timorense
Saccharum_hybrid_cv._NCo_310
Imperata_cylindrica
Sorghastrum_nutans
Eulalia_aurea
Capillipedium_venustrum
Bothriochloa_alta
Iseilema_macratherum
Themeda_sp.
Hyparrhenia_subplumosa
Diheteropogon_amplectans_var._catangensis
Rottboellia_cochinchinensis
Coix_lacryma−jobi
Ischaemum_afrum
Zea_mays_subsp._mays
Arthraxon_prionodes
Arundinella_deppeana
Steinchisma_laxum
Otachyrium_versicolor
Plagiantha_tenella
Oncorachis_ramosa
Coleataenia_prionitis
Paspalum_fimbriatum
Paspalum_dilatatum
Paspalum_glazovii
Axonopus_fissifolius
Setaria_viridis
Setaria_italica
Cenchrus_americanus
Setaria_geminata
Urochloa_reptans
Eriochloa_meyeriana
Megathrysus_maximus
Whiteochloa_capillipes
Panicum_capillare
Panicum_virgatum
Dichanthelium_acuminatum
Thyridolepis_xerophila
Alloteropsis_semialata
Alloteropsis_angusta
Alloteropsis_cimicina
Amphicarpum_muhlenbergianum
Echinochloa_crus−galli
Echinochloa_oryzicola
Oplismenus_hirtellus
Digitaria_exilis
Lecomtella_madagascariensis
Loudetiopsis_kerstingii
Danthoniopsis_dinteri
Centotheca_lappacea
Thysanolaena_latifolia
Zeugites_pittieri
Chasmanthium_sessiliflorum
Stipagrostis_uniplumis_var._uniplumis
Stipagrostis_hirtigluma
Sartidia_perrieri
Sartidia_dewinteri
Aristida_purpurea
Aristida_ternipes
Oxychloris_scariosa
Eustachys_glauca
Chloris_barbata
Leptochloa_pluriflora
Astrebla_pectinata
Trichoneura_grandiglumis
Dactyloctenium_aegyptium
Triodia_stipoides
Triodia_wiseana
Melanocenchris_abyssinica
Halopyrum_mucronatum
Bouteloua_curtipendula
Bouteloua_gracilis
Distichlis_spicata
Distichlis_bajaensis
Hilaria_cenchroides
Hilaria_rigida
Sporobolus_maritimus
Sporobolus_michauxianus
Sporobolus_heterolepis
Zoysia_macrantha
Eragrostis_tef
Eragrostis_minor
Uniola_paniculata
Neyraudia_reynaudiana
Centropodia_glauca
Rytidosperma_semiannulare
Rytidosperma_pallidum
Tenaxia_guillarmodiae
Tribolium_hispidum
Danthonia_californica
Chaetobromus_involucratus_subsp._dregeanus
Chionochloa_macra
Phragmites_australis
Hakonechloa_macra
Elytrophorus_spicatus
Monachather_paradoxa
Eriachne_stipacea
Isachne_distichophylla
Micraira_spiciformis
Puella_olyriformis
Leptaspis_zeylanica
Leptaspis_banksii
Pharus_latifolius
Pharus_lappulaceus
Streptochaeta_spicata
Anomochloa_marantoidea
0.01

H

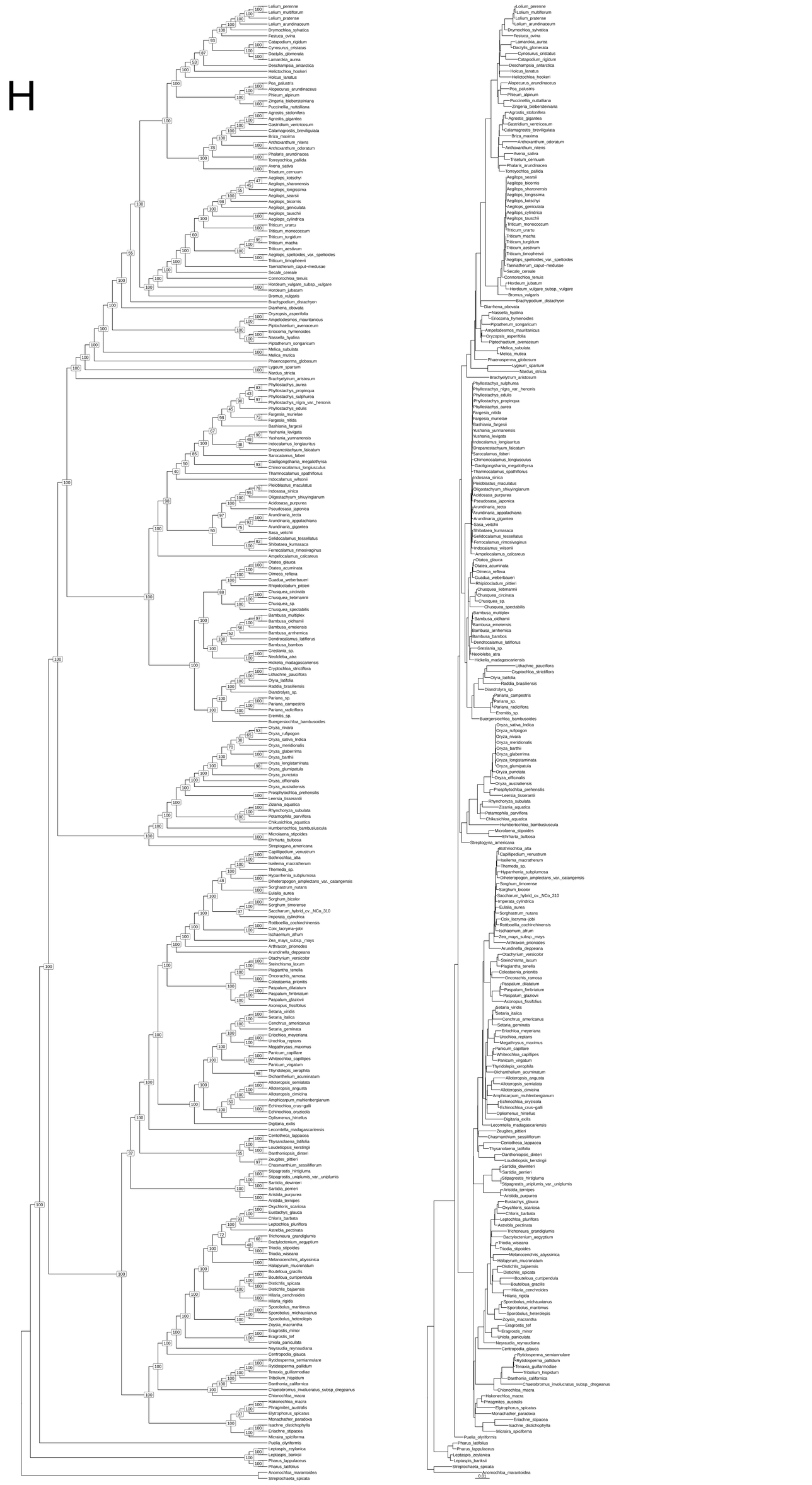
Lolium_perenne
Lolium_multiflorum
Lolium_pratense
Lolium_arundinaceum
Drymochloa_sylvatica
Festuca_ovina
Catapodium_rigidum
Cynosurus_cristatus
Dactylis_glomerata
Lamarckia_aurea
Deschampsia_antarctica
Helictochloa_hookeri
Holcus_lanatus
Poa_palustris
Alopecurus_arundinaceus
Phleum_alpinum
Zingeria_biebersteiniana
Puccinellia_nuttalliana
Agrostis_stolonifera
Agrostis_gigantea
Gastridium_ventricosum
Calamagrostis_brevilig ulata
Briza_maxima
Anthoxanthum_nitens
Anthoxanthum_odoratum
Phalaris_arundinacea
Torreyochloa_pallida
Avena_sativa
Trisetum_cernuum
Aegilops_kotschyi
Aegilops_sharonensis
Aegilops_longissima
Aegilops_searsii
Aegilops_bicornis
Aegilops_geniculata
Aegilops_tauschii
Aegilops_cylindrica
Triticum_urartu
Triticum_monococcum
Triticum_turgidum
Triticum_macha
Triticum_aestivum
Aegilops_speltoides_var._speltoides
Triticum_timopheevii
Taeniatherum_caput-medusae
Secale_cereale
Connorochloa_tenuis
Hordeum_vulgare_subsp._vulgare
Hordeum_jubatum
Bromus_vulgaris
Brachypodium_distachyon
Diarrhena_obovata
Oryzopsis_asperifolia
Ampelodesmos_mauritanicus
Piptochaetium_avenaceum
Eriocoma_hymenoides
Nassella_hyalina
Piptatherum_songaricum
Melica_subulata
Melica_mutica
Phaenosperma_globosum
Lygeum_spartum
Nardus_stricta
Brachyelytrum_aristosum
Phyllostachys_aurea
Phyllostachys_propinqua
Phyllostachys_sulphurea
Phyllostachys_nigra_var._henonis
Phyllostachys_edulis
Fargesia_murielae
Fargesia_nitida
Bashania_fargesii
Yushania_levigata
Yushania_yunnanensis
Indocalamus_longiauritus
Drepanostachyum_falcatum
Sarocalamus_faberi
Gaoligongshania_megalothyrsa
Chimonocalamus_longiusculus
Thamnocalamus_spathiflorus
Indocalamus_wilsonii
Pleioblastus_maculatus
Indosasa_sinica
Oligostachyum_shiuyingianum
Acidosasa_purpurea
Pseudosasa_japonica
Arundinaria_tecta
Arundinaria_appalachiana
Arundinaria_gigantea
Sasa_veitchii
Shibataea_kumasaca
Gelidocalamus_tessellatus
Ferrocalamus_rimosivaginus
Ampelocalamus_calcareus
Otatea_glauca
Otatea_acuminata
Olmeca_reflexa
Guadua_weberbaueri
Rhipidocladum_pittieri
Chusquea_liebmannii
Chusquea_circinata
Chusquea_sp.
Chusquea_spectabilis
Bambusa_multiplex
Bambusa_oldhamii
Bambusa_emeiensis
Bambusa_arnhemica
Bambusa_bambos
Dendrocalamus_latiflorus
Greslania_sp.
Neololeba_atra
Hickelia_madagascariensis
Cryptochloa_strictiflora
Lithachne_pauciflora
Olyra_latifolia
Raddia_brasiliensis
Diandrolyra_sp.
Pariana_sp.
Pariana_campestris
Pariana_radiciflora
Eremitis_sp.
Buergersiochloa_bambusoides
Oryza_nivara
Oryza_rufipogon
Oryza_sativa_Indica
Oryza_meridionalis
Oryza_glaberrima
Oryza_barthii
Oryza_longistaminata
Oryza_glumipatula
Oryza_punctata
Oryza_officinalis
Oryza_australiensis
Prosphytochloa_prehensilis
Leersia_tisserantii
Zizania_aquatica
Rhynchoryza_subulata
Potamophila_parviflora
Chikusichloa_aquatica
Humbertochloa_bambusiuscula
Microlaena_stipoides
Ehrharta_bulbosa
Streptogyna_americana
Capillipedium_venustrum
Bothriochloa_alta
Iseilema_macratherum
Themeda_sp.
Hyparrhenia_subplumosa
Diheteropogon_amplectans_var._catangensis
Sorghastrum_nutans
Eulalia_aurea
Sorghum_bicolor
Sorghum_timorense
Saccharum_hybrid_cv._NCo_310
Imperata_cylindrica
Rottboellia_cochinchinensis
Coix_lacryma-jobi
Ischaemum_afrum
Zea_mays_subsp._mays
Arthraxon_prionodes
Arundinella_deppeana
Otachyrium_versicolor
Steinchisma_laxum
Plagiantha_tenella
Oncorachis_ramosa
Coleataenia_prionitis
Paspalum_dilatatum
Paspalum_fimbriatum
Paspalum_glazovii
Axonopus_fissifolius
Setaria_viridis
Setaria_italica
Cenchrus_americanus
Setaria_geminata
Eriochloa_meyeriana
Urochloa_reptans
Megathrysus_maximus
Panicum_capillare
Whiteochloa_capillipes
Panicum_virgatum
Thyridolepis_xerophila
Dichanthelium_acuminatum
Alloteropsis_semialata
Alloteropsis_angusta
Alloteropsis_cimicina
Amphicarpum_muhlenbergianum
Echinochloa_crus-galli
Echinochloa_oryzicola
Oplismenus_hirtellus
Digitaria_exilis
Lecomtella_madagascariensis
Centotheca_lappacea
Thysanolaena_latifolia
Loudetopsis_kerstingii
Danthoniopsis_dinteri
Zeugites_pittieri
Chasmanthium_sessiliflorum
Stipagrostis_hirtigluma
Stipagrostis_uniplumis_var._uniplumis
Sartidia_dewinteri
Sartidia_perrieri
Aristida_purpurea
Aristida_ternipes
Oxychloris_scariosa
Eustachys_glauca
Chloris_barbata
Leptochloa_pluriflora
Astrebla_pectinata
Trichoneura_grandiglumis
Dactyloctenium_aegyptium
Triodia_stipoides
Triodia_wiseana
Melanocenchris_abyssinica
Halopyrum_mucronatum
Boutelloua_gracilis
Boutelloua_curtipendula
Distichlis_spicata
Distichlis_bajaensis
Hilaria_cenchroides
Hilaria_rigida
Sporobolus_maritimus
Sporobolus_michauxianus
Sporobolus_heterolepis
Zoysia_macrantha
Eragrostis_tef
Eragrostis_minor
Eragrostis_tef
Uniola_paniculata
Neyraudia_reynaudiana
Centropodia_glauca
Rytidosperma_semiannulare
Rytidosperma_pallidum
Tenaxia_guillarmodiae
Tribolium_hispidum
Danthonia_californica
Chaetobromus_involucratus_subsp._dregeanus
Chionochloa_macra
Hakonechloa_macra
Phragmites_australis
Elytrophorus_spicatus
Monachather_paradoxa
Isachne_distichophylla
Eriachne_stipacea
Micraira_spiciformis
Puella_olyriformis
Leptaspis_zeylanica
Leptaspis_banksii
Pharus_latifolius
Pharus_lappulaceus
Anomochloa_marantoidea
Streptochaeta_spicata
0.01

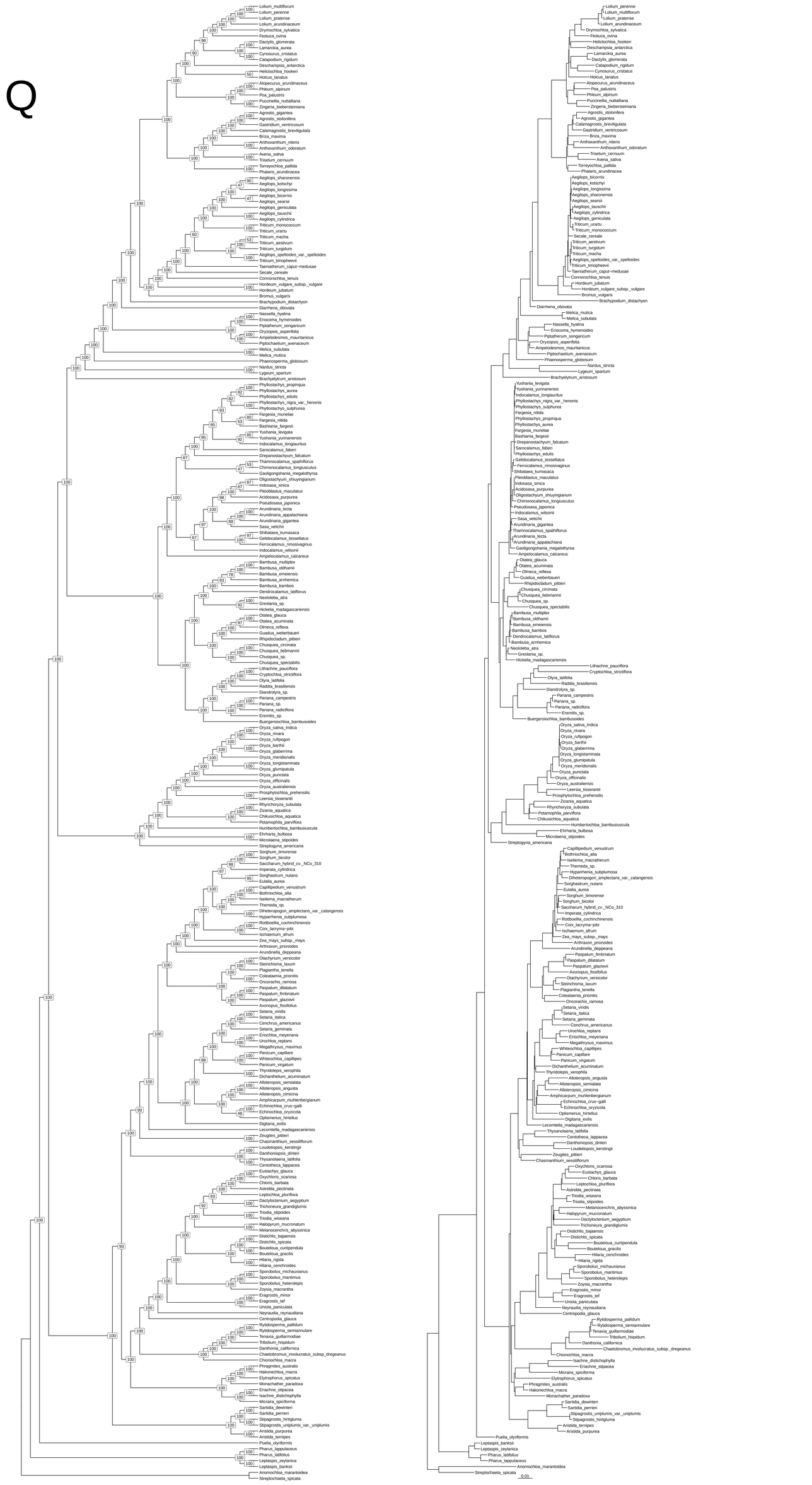
Q
Lolium_multiflorum
Lolium_perenne
Lolium_pratense
Lolium_arundinaceum
Drymochloa_sylvatica
Festuca_ovina
Dactylis_glomerata
Lamarckia_aurea
Cynosurus_cristatus
Catapodium_rigidum
Deschampsia_antarctica
Helictochloa_hookeri
Holcus_lanatus
Alopecurus_arundinaceus
Phleum_alpinum
Poa_palustris
Puccinellia_nuttalliana
Zingeria_biebersteiniana
Agrostis_gigantea
Agrostis_stolonifera
Gastridium_ventricosum
Calamagrostis_breviligulata
Briza_maxima
Anthoxanthum_nitens
Anthoxanthum_odoratum
Avena_sativa
Trisetum_cernuum
Torreyochloa_pallida
Phalaris_arundinacea
Aegilops_sharonensis
Aegilops_kotschyi
Aegilops_longissima
Aegilops_bicornis
Aegilops_searsii
Aegilops_geniculata
Aegilops_tauschii
Aegilops_cylindrica
Triticum_monococcum
Triticum_urartu
Triticum_macha
Triticum_aestivum
Triticum_turgidum
Aegilops_speltoides_var._speltoides
Triticum_timopheevii
Taeniatherum_caput-medusae
Secale_cereale
Connorochloa_tenuis
Hordeum_vulgare_subsp._vulgare
Hordeum_jubatum
Bromus_vulgaris
Brachypodium_distachyon
Diarrhena_obovata
Nassella_hyalina
Eriocoma_hymenoides
Piptatherum_songaricum
Oryzopsis_asperifolia
Ampelodesmos_mauritanicus
Piptochaetium_avenaceum
Melica_subulata
Melica_mutica
Phaenosperma_globosum
Nardus_stricta
Lygeum_spartum
Brachyelytrum_aristosum
Yushania_levigata
Yushania_yunnanensis
Indocalamus_longiauritus
Phyllostachys_nigra_var._henonis
Phyllostachys_sulphurea
Fargesia_nitida
Fargesia_murielae
Phyllostachys_propinqua
Phyllostachys_aurea
Bashiania_fargesii
Drepanostachyum_falcatum
Sarocalamus_faberi
Phyllostachys_edulis
Gelidocalamus_tessellatus
Ferrocalamus_rimosivaginus
Shibataea_kumasaca
Pleioblastus_maculatus
Indosasa_sinica
Acidosasa_purpurea
Oligostachyum_shiuyingianum
Chimonocalamus_longiusculus
Pseudosasa_japonica
Indocalamus_wilsonii
Sasa_veitchii
Arundinaria_gigantea
Arundinaria_tecta
Arundinaria_appalachiana
Gaoligongshania_megalotrhyrsa
Ampelocalamus_calcareus
Otatea_glauca
Otatea_acuminata
Olmeca_reflexa
Guadua_weberbaueri
Rhipidocladum_pittieri
Chusquea_circinata
Chusquea_liebmannii
Chusquea_sp.
Chusquea_spectabilis
Bambusa_multiplex
Bambusa_oldhamii
Bambusa_emeiensis
Bambusa_bambos
Dendrocalamus_latiflorus
Bambusa_arnhemica
Neololeba_atra
Greslania_sp.
Hickelia_madagascariensis
Lithachne_pauciflora
Cryptochloa_strictiflora
Olyra_latifolia
Raddia_brasiliensis
Diandrolyra_sp.
Pariana_campestris
Pariana_sp.
Pariana_radiciflora
Eremitis_sp.
Buergersiochloa_bambusoides
Oryza_sativa_Indica
Oryza_nivara
Oryza_rufipogon
Oryza_barthii
Oryza_glaberrima
Oryza_longistaminata
Oryza_glumipatula
Oryza_meridionalis
Oryza_punctata
Oryza_officinalis
Oryza_australiensis
Leersia_tisseranti
Prosphytochloa_prehensilis
Zizania_aquatica
Rhynchoryza_subulata
Chikusichloa_aquatica
Phragmites_australis
Hakonechloa_macra
Monachather_paradoxa
Sartidia_dewinteri
Sartidia_perrieri
Stipagrostis_uniplumis_var._uniplumis
Stipagrostis_hirtigluma
Aristida_purpurea
Aristida_ternipes
Humbertochloa_bambusiuscula
Ehrharta_bulbosa
Microlaena_stipoides
Streptogyna_americana
Capillipedium_venustrum
Bothriochloa_alta
Iseilema_macratherum
Themeda_sp.
Hyparrhenia_subplumosa
Dheteropogon_amplectans_var._catangensis
Sorghastrum_nutans
Eulalia_aurea
Sorghum_timorense
Sorghum_bicolor
Saccharum_hybrid_cv._NCo_310
Imperata_cylindrica
Rottboellia_cochinchinensis
Coix_lacryma-jobi
Ischaemum_afrum
Zea_mays_subsp._mays
Arthraxon_prionodes
Arundinella_deppeana
Paspalum_fimbriatum
Paspalum_dilatatum
Paspalum_glaziovii
Axonopus_fissifolius
Otachyrium_versicolor
Steinchisma_laxum
Plagiantha_tenella
Coleataenia_prionitis
Oncorachis_ramosa
Setaria_viridis
Setaria_italica
Setaria_geminata
Cenchrus_americanus
Urochloa_reptans
Eriochloa_meyeriana
Megathrysus_maximus
Whiteochloa_capillipes
Panicum_capillare
Panicum_virgatum
Dichanthelium_acuminatum
Thyridolepis_xerophila
Alloteropsis_angusta
Alloteropsis_semialata
Alloteropsis_cimicina
Amphicarpum_muhlenbergianum
Echinochloa_crus-galli
Echinochloa_oryzicola
Oplismenus_hirtellus
Digitaria_exilis
Lecomtella_madagascariensis
Thysanolaena_latifolia
Centotheca_lappacea
Danthoniopsis_dinteri
Loudetiopsis_kerstingii
Zeugites_pittieri
Chasmanthium_sessiliflorum
Oxychloris_scariosa
Eustachys_glauca
Chloris_barbata
Leptochloa_pluriflora
Astrebla_pectinata
Triodia_wiseana
Triodia_stipoides
Melanocenchris_abyssinica
Halopyrum_mucronatum
Dactyloctenium_aegyptium
Trichoneura_grandiglumis
Distichlis_bajaensis
Distichlis_spicata
Bouteloua_curtipendula
Bouteloua_gracilis
Hilaria_rigida
Hilaria_cenchroides
Sporobolus_michauxianus
Sporobolus_maritimus
Sporobolus_heterolepis
Zoysia_macrantha
Eragrostis_minor
Eragrostis_tef
Uniola_paniculata
Neyraudia_reynaudiana
Centropodia_glauca
Rytidosperma_pallidum
Rytidosperma_semiannulare
Tenaxia_guillarmodiae
Tribolium_hispidum
Danthonia_californica
Chaetobromus_involucratus_subsp._dregeanus
Chionochloa_macra
Isachne_distichophylla
Eriachne_stipacea
Micraira_spiciformis
Elytrophorus_spicatus
Puelia_olyriformis
Leptaspis_banksii
Leptaspis_zeylanica
Pharus_latifolius
Pharus_lappulaceus
Anomochloa_marantoidea
Streptochaeta_spicata
0.01

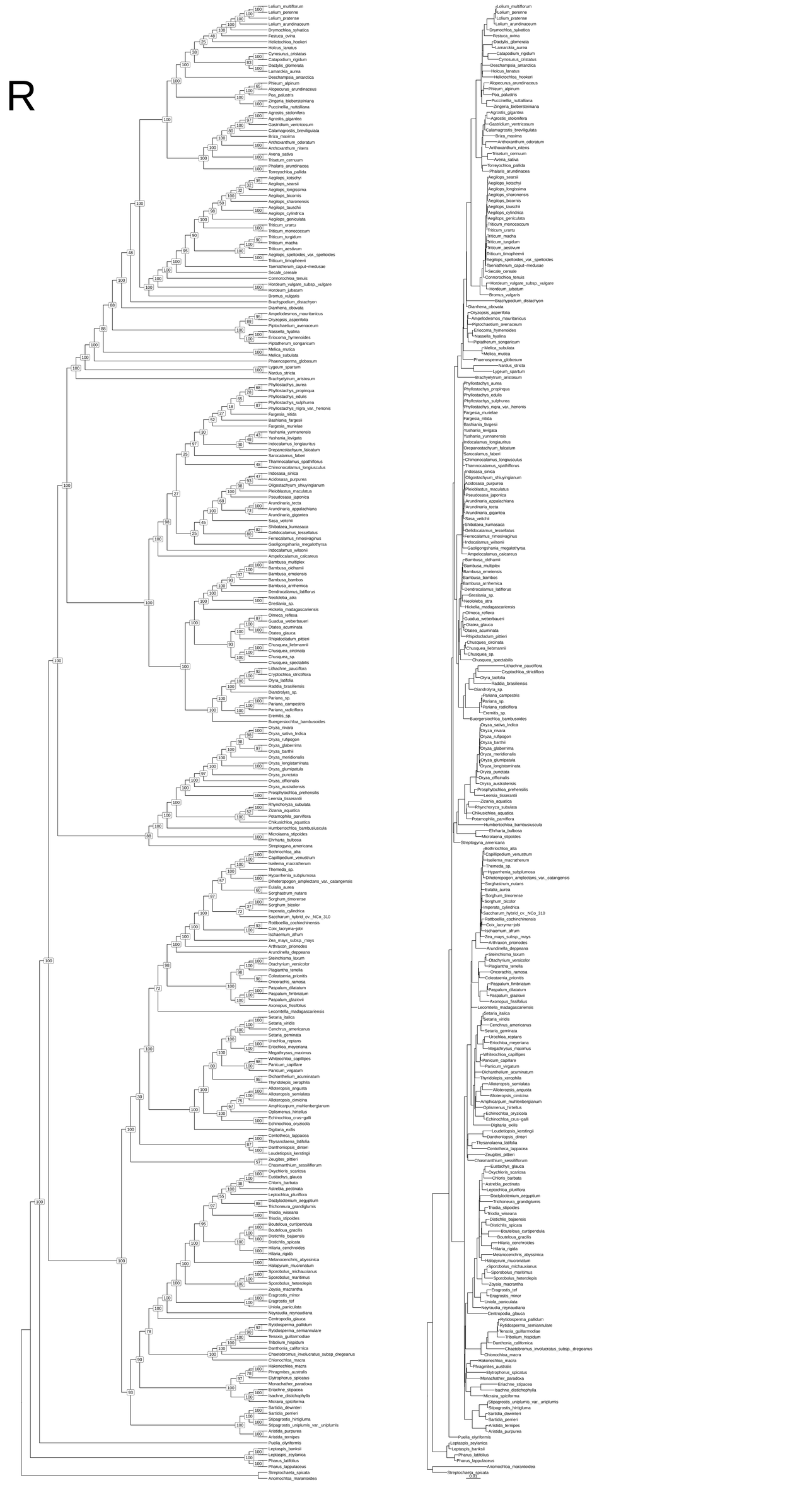
R
Lolium_multiflorum
Lolium_perenne
Lolium_pratense
Lolium_arundinaceum
Drymochloa_sylvatica
Festuca_ovina
Dactylis_glomerata
Lamarckia_aurea
Catapodium_rigidum
Cynosurus_cristatus
Deschampsia_antarctica
Holcus_lanatus
Helictochloa_hookeri
Alopecurus_arundinaceus
Phleum_alpinum
Poa_palustris
Puccinellia_nuttalliana
Zingeria_biebersteiniana
Agrostis_gigantea
Agrostis_stolonifera
Gastridium_ventricosum
Calamagrostis_breviligulata
Briza_maxima
Anthoxanthum_odoratum
Anthoxanthum_nitens
Trisetum_cernuum
Avena_sativa
Torreyochloa_pallida
Phalaris_arundinacea
Aegilops_kotschyi
Aegilops_searsii
Aegilops_longissima
Aegilops_sharonensis
Aegilops_biuncialis
Aegilops_tauschii
Aegilops_cylindrica
Aegilops_geniculata
Triticum_urartu
Triticum_monococcum
Triticum_turgidum
Triticum_macha
Triticum_aestivum
Triticum_timopheevii
Aegilops_speltoides_var._speltoides
Taeniatherum_caput-medusae
Secale_cereale
Connorochloa_tenuis
Hordeum_vulgare_subsp._vulgare
Hordeum_jubatum
Bromus_vulgaris
Brachypodium_distachyon
Diarrhena_obovata
Ampelodesmos_mauritanicus
Oryzopsis_asperifolia
Piptochaetium_avenaceum
Nassella_hyalina
Eriocoma_hymenoides
Piptatherum_songaricum
Melica_mutica
Melica_subulata
Phaenosperma_globosum
Lygeum_spartum
Nardus_stricta
Brachyelytrum_aristosum
Phyllostachys_aurea
Phyllostachys_propinqua
Phyllostachys_edulis
Phyllostachys_sulphurea
Phyllostachys_nigra_var._henonis
Fargesia_nitida
Bashania_fargesii
Fargesia_murielae
Yushania_yunnanensis
Yushania_levigata
Indocalamus_longiauritus
Drepanostachyum_falcatum
Sarocalamus_faberi
Thamnocalamus_spathiflorus
Chimonocalamus_longiusculus
Indosasa_sinica
Acidosasa_purpurea
Oligostachyum_shuyingianum
Pleioblastus_maculatus
Pseudosasa_japonica
Arundinaria_tecta
Arundinaria_appalachiana
Arundinaria_gigantea
Sasa_veitchii
Shibataea_kumasaca
Gelidocalamus_tessellatus
Ferrocalamus_rimosivaginus
Gaoligongshania_megalothyrsa
Indocalamus_wilsonii
Ampelocalamus_calcareus
Bambusa_multiplex
Bambusa_oldhamii
Bambusa_emeiensis
Bambusa_bambos
Bambusa_arnhemica
Dendrocalamus_latiflorus
Neololeba_atra
Greslania_sp.
Hickelia_madagascariensis
Olmeca_reflexa
Guadua_weberbaueri
Otatea_acuminata
Otatea_glauca
Rhipidocladum_pittieri
Chusquea_liebmannii
Chusquea_circinata
Chusquea_sp.
Chusquea_spectabilis
Lithachne_pauciflora
Cryptochloa_strictiflora
Olyra_latifolia
Raddia_brasiliensis
Diandrolyra_sp.
Pariana_campestris
Pariana_sp.
Pariana_radiciflora
Eremitis_sp.
Buergersiochloa_bambusoides
Oryza_nivara
Oryza_sativa_Indica
Oryza_rufipogon
Oryza_glaberrima
Oryza_barthii
Oryza_meridionalis
Oryza_longistaminata
Oryza_glumipatula
Oryza_punctata
Oryza_officinalis
Oryza_australiensis
Prosphytochloa_prehensilis
Leersia_tisserantii
Rhynchoryza_subulata
Zizania_aquatica
Potamophila_parviflora
Chikusichloa_aquatica
Humbertochloa_bambusiuscula
Microlaena_stipoides
Ehrharta_bulbosa
Streptogyna_americana
Bothriochloa_alta
Capillipedium_venustrum
Iseilema_macratherum
Themeda_sp.
Hyparrhenia_subplumosa
Diheteropogon_amplectans_var._catangensis
Eulalia_aurea
Sorghastrum_nutans
Sorghum_timorense
Sorghum_bicolor
Imperata_cylindrica
Saccharum_hybrid_cv._NCo_310
Rottboellia_cochinchinensis
Coix_lacryma-jobi
Ischaemum_afrum
Zea_mays_subsp._mays
Arthraxon_prionodes
Arundinella_deppeana
Steinchisma_laxum
Otachyrium_versicolor
Plagiantha_tenella
Coleataenia_prionitis
Oncorachis_ramosa
Paspalum_dilatatum
Paspalum_fimbriatum
Paspalum_glaziovii
Axonopus_fissifolius
Lecomtella_madagascariensis
Setaria_italica
Setaria_viridis
Cenchrus_americanus
Setaria_geminata
Urochloa_reptans
Eriochloa_meyeriana
Megathyrsus_maximus
Whiteochloa_capillipes
Panicum_capillare
Panicum_virgatum
Dichanthelium_acuminatum
Thyridolepis_xerophila
Alloteropsis_angusta
Alloteropsis_semialata
Alloteropsis_cimicina
Amphicarpum_muhlenbergianum
Oplismenus_hirtellus
Echinochloa_crus-galli
Echinochloa_oryzicola
Digitaria_exilis
Centotheca_lappacea
Thysanolaena_latifolia
Danthoniopsis_dinteri
Loudetopsis_kerstingii
Zeugites_pittieri
Chasmanthium_sessiliflorum
Oxychloris_scariosa
Eustachys_glauca
Chloris_barbata
Astrebla_pectinata
Leptochloa_plurriflora
Dactyloctenium_aegyptium
Trichoneura_grandiglumis
Triodia_wiseana
Triodia_stipoides
Bouteloua_curtipendula
Bouteloua_gracilis
Distichlis_bajaensis
Distichlis_spicata
Hilaria_cenchroides
Hilaria_rigida
Melanocenchris_abyssinica
Halopyrum_mucronatum
Sporobolus_michauxianus
Sporobolus_maritimus
Sporobolus_heterolepis
Zoysia_macrantha
Eragrostis_minor
Eragrostis_tef
Uniola_paniculata
Neyraudia_reynaudiana
Centropodia_glauca
Rytidosperma_pallidum
Rytidosperma_semiannulare
Tenaxia_guillarmodiae
Tribolium_hispidum
Danthonia_californica
Chaetobromus_involucratus_subsp._dregeanus
Chionochloa_macra
Hakonechloa_macra
Phragmites_australis
Elytrophorus_spicatus
Monachather_paradoxa
Eriachne_stipacea
Isachne_distichophylla
Micraira_spiciformis
Sartidia_dewinteri
Sartidia_perrieri
Stipagrostis_hirtigluma
Stipagrostis_uniplumis_var._uniplumis
Aristida_purpurea
Aristida_ternipes
Puella_olyriformis
Leptaspis_banksii
Leptaspis_zeylanica
Pharus_latifolius
Pharus_lappulaceus
Streptochaeta_spicata
Anomochloa_marantoidea
0.01

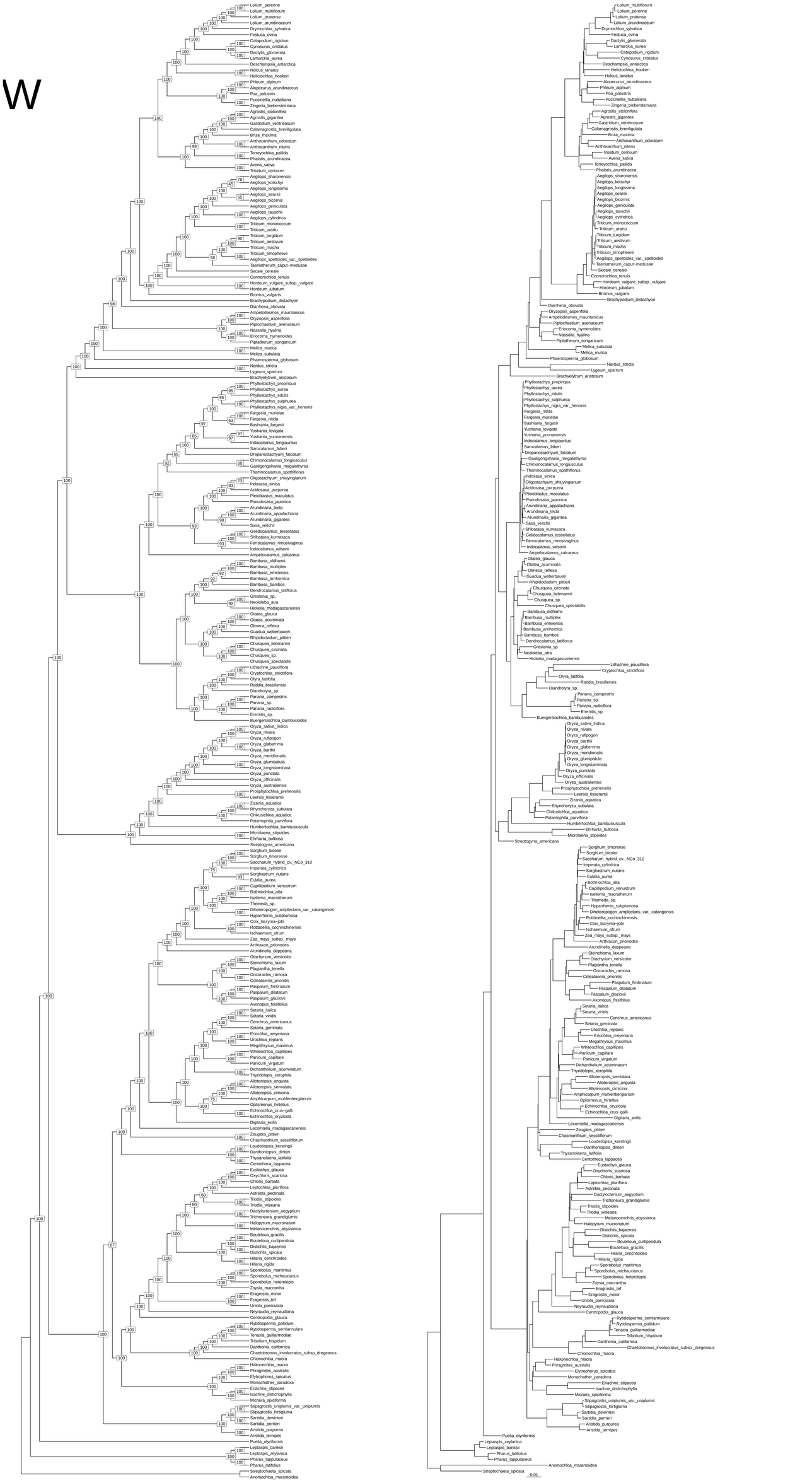
Lolium_perenne
Lolium_multiflorum
Lolium_pratense
Lolium_arundinaceum
Drymochloa_sylvatica
Festuca_ovina
Catapodium_rigidum
Cynosurus_cristatus
Dactylis_glomerata
Lamarckia_aurea
Deschampsia_antarctica
Holcus_lanatus
Helictochloa_hookeri
Phleum_alpinum
Alopecurus_arundinaceus
Poa_palustris
Puccinellia_nuttalliana
Zingeria_biebersteiniana
Agrostis_stolonifera
Agrostis_gigantea
Gastridium_ventricosum
Calamagrostis_brevilygulata
Briza_maxima
Anthoxanthum_odoratum
Anthoxanthum_nitens
Torreyochloa_pallida
Phalaris_arundinacea
Avena_sativa
Trisetum_cernuum
Aegilops_sharonensis
Aegilops_kotschyi
Aegilops_longissima
Aegilops_searsii
Aegilops_bicornis
Aegilops_geniculata
Aegilops_tauschii
Aegilops_cylindrica
Triticum_monococcum
Triticum_urartu
Triticum_turgidum
Triticum_aestivum
Triticum_macha
Triticum_timopheevii
Aegilops_speltoides_var._speltoides
Taeniatherum_caput-medusae
Secale_cereale
Connorochloa_tenuis
Hordeum_vulgare_subsp._vulgare
Hordeum_jubatum
Bromus_vulgaris
Brachypodium_distachyon
Diarrhena_obovata
Ampelodesmos_mauritanicus
Oryzopsis_asperifolia
Piptochaetium_avenaceum
Nassella_hyalina
Eriocoma_hymenoides
Piptatherum_songaricum
Melica_mutica
Melica_subulata
Phaenosperma_globosum
Nardus_stricta
Lygeum_spartum
Brachyelytrum_aristosum
Phyllostachys_propinqua
Phyllostachys_aurea
Phyllostachys_edulis
Phyllostachys_sulphurea
Phyllostachys_nigra_var._henonis
Fargesia_murielae
Fargesia_nitida
Bashania_fargesii
Yushania_levigata
Yushania_yunnanensis
Indocalamus_longiauritus
Sarocalamus_faberi
Drepanostachyum_falcatum
Gaoligongshania_megalothyrsa
Chimonocalamus_longiusculus
Thamnocalamus_spathiflorus
Oligostachyum_shiuyinginanum
Indosasa_sinica
Acidosasa_purpurea
Pleioblastus_maculatus
Pseudosasa_japonica
Arundinaria_tecta
Arundinaria_appalachiana
Arundinaria_gigantea
Sasa_veitchii
Shibataea_kumasaca
Gelidocalamus_tessellatus
Ferrocalamus_rimosivaginus
Indocalamus_wilsonii
Ampelocalamus_calcareus
Bambusa_oldhamii
Bambusa_multiplex
Bambusa_emeiensis
Bambusa_arnhemica
Bambusa_bambos
Dendrocalamus_latiflorus
Greslania_sp.
Neololeba_atra
Hickelia_madagascariensis
Otatea_glauca
Otatea_acuminata
Olmeca_reflexa
Guadua_weberbaueri
Rhipidocladum_pittieri
Chusquea_liebmannii
Chusquea_circinata
Chusquea_sp.
Chusquea_spectabilis
Lithachne_pauciflora
Cryptochloa_strictiflora
Olyra_latifolia
Raddia_brasiliensis
Diandrolyra_sp.
Pariana_campestris
Pariana_sp.
Pariana_radiciflora
Eremitis_sp.
Buergersiochloa_bambusoides
Oryza_sativa_indica
Oryza_nivara
Oryza_rufipogon
Oryza_glaberrima
Oryza_barthii
Oryza_meridionalis
Oryza_glumipatula
Oryza_longistaminata
Oryza_punctata
Oryza_officinalis
Oryza_australiensis
Prosphytochloa_prehensilis
Leersia_tisserantii
Zizania_aquatica
Rhynchoryza_subulata
Chikusichloa_aquatica
Potamophila_parviflora
Humbertochloa_bambusiuscula
Microlaena_stipoides
Ehrharta_bulbosa
Streptogyna_americana
Sorghum_bicolor
Sorghum_timorense
Saccharum_hybrid_cv._NCo_310
Imperata_cylindrica
Sorghastrum_nutans
Eulalia_aurea
Capillipedium_venustrum
Bothriochloa_alta
Iseilema_macratherum
Themeda_sp.
Diheteropogon_amplectans_var._catangensis
Hyparrhenia_subplumosa
Coix_lacryma-jobi
Rottboellia_cochinchinensis
Ischaemum_afrum
Zea_mays_subsp._mays
Arthraxon_prionodes
Arundinella_deppeana
Otachyrium_versicolor
Steinchisma_laxum
Plagiantha_tenella
Oncorachis_ramosa
Coleataenia_prionitis
Paspalum_fimbriatum
Paspalum_dilatatum
Paspalum_glazovii
Axonopus_fissifolius
Setaria_italica
Setaria_viridis
Cenchrus_americanus
Setaria_geminata
Eriochloa_meyeriana
Urochloa_reptans
Megathrysus_maximus
Whiteochloa_capillipes
Panicum_capillare
Panicum_virgatum
Dichanthelium_acuminatum
Thyridolepis_xerophila
Alloteropsis_angusta
Alloteropsis_semialata
Alloteropsis_cimicina
Amphicarpum_muhlenbergianum
Oplismenus_hirtellus
Echinochloa_crus-galli
Echinochloa_oryzicola
Digitaria_exilis
Lecomtella_madagascariensis
Zeugites_pittieri
Chasmanthium_sessiliflorum
Loudetiopsis_kerstingii
Danthoniopsis_dinteri
Thysanolaena_latifolia
Centotheca_lappacea
Eustachys_glauca
Oxychloris_scariosa
Chloris_barbata
Leptochloa_pluriflora
Astrebla_pectinata
Triodia_stipoides
Triodia_wiseana
Dactyloctenium_aegyptium
Trichoneura_grandiglumis
Halopyrum_mucronatum
Melanocenchris_abyssinica
Bouteloua_gracilis
Bouteloua_curtipendula
Distichlis_bajaensis
Distichlis_spicata
Hilaria_cenchroides
Hilaria_rigida
Sporobolus_maritimus
Sporobolus_michauxianus
Sporobolus_heterolepis
Zoysia_macrantha
Eragrostis_minor
Eragrostis_tef
Uniola_paniculata
Neyraudia_reynaudiana
Centropodia_glauca
Rytidosperma_pallidum
Rytidosperma_semiannulare
Tenaxia_guillarmodiae
Tribolium_hispidum
Danthonia_californica
Chaetobromus_involucratus_subsp._dregeanus
Chionochloa_macra
Hakonechloa_macra
Phragmites_australis
Elytrophorus_spicatus
Monachather_paradoxa
Eriachne_stipacea
Isachne_distichophylla
Micraira_spiciformis
Stipagrostis_uniplumis_var._uniplumis
Stipagrostis_hirtigluma
Sartidia_dewinteri
Sartidia_perrieri
Aristida_purpurea
Aristida_ternipes
Puella_olyriformis
Leptaspis_banksii
Leptaspis_zeylanica
Pharus_latifolius
Pharus_lappulaceus
Anomochloa_marantoidea
Streptochaeta_spicata
0.01

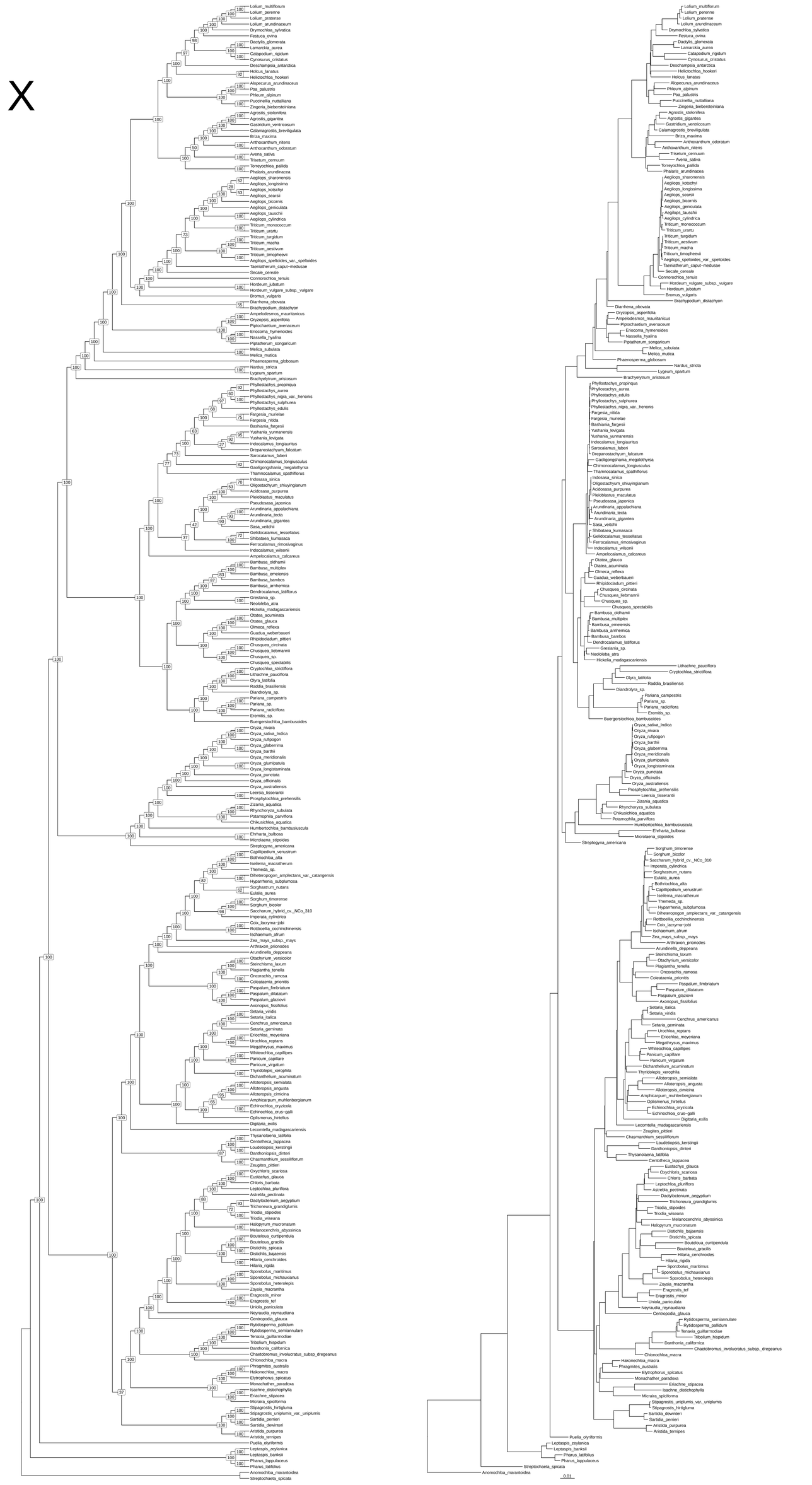
X
Lolium_multiflorum
Lolium_perenne
Lolium_pratense
Lolium_arundinaceum
Drymochloa_sylvatica
Festuca_ovina
Dactylis_glomerata
Lamarckia_aurea
Catapodium_rigidum
Cynosurus_cristatus
Deschampsia_antarctica
Holcus_lanatus
Helictochloa_hookeri
Alopecurus_arundinaceus
Poa_palustris
Phleum_alpinum
Puccinellia_nuttalliana
Zingeria_biebersteiniana
Agrostis_stolonifera
Agrostis_gigantea
Gastridium_ventricosum
Calamagrostis_breviligulata
Briza_maxima
Anthoxanthum_nitens
Anthoxanthum_odoratum
Avena_sativa
Trisetum_cernuum
Torreyochloa_pallida
Phalaris_arundinacea
Aegilops_sharonensis
Aegilops_longissima
Aegilops_kotschyi
Aegilops_searsii
Aegilops_bicornis
Aegilops_geniculata
Aegilops_tauschii
Aegilops_cylindrica
Triticum_monococcum
Triticum_urartu
Triticum_turgidum
Triticum_aestivum
Triticum_timopheevii
Aegilops_speltoides_var._speltoides
Taeniatherum_caput-medusae
Secale_cereale
Connorochloa_tenuis
Hordeum_jubatum
Hordeum_vulgare_subsp._vulgare
Bromus_vulgaris
Diarrhena_obovata
Brachypodium_distachyon
Ampelodesmos_mauritanicus
Oryzopsis_asperifolia
Piptochaetium_avenaceum
Eriocoma_hymenoides
Nassella_hyalina
Piptatherum_songaricum
Melica_subulata
Melica_mutica
Phaenosperma_globosum
Nardus_stricta
Lygeum_spartum
Brachyelytrum_aristosum
Phyllostachys_propinqua
Phyllostachys_aurea
Phyllostachys_nigra_var._henonis
Phyllostachys_sulphurea
Phyllostachys_edulis
Fargesia_nitida
Fargesia_murielae
Bashania_fargesii
Yushania_levigata
Yushania_yunnanensis
Indocalamus_longiauritus
Drepanostachyum_falcatum
Gaoligongshania_megalothyrsa
Chimonocalamus_longiusculus
Thamnocalamus_spathiflorus
Indosasa_sinica
Oligostachyum_shiuyingianum
Acidosasa_purpurea
Pleioblastus_maculatus
Pseudosasa_japonica
Arundinaria_appalachiana
Arundinaria_tecta
Arundinaria_gigantea
Sasa_veitchii
Shibataea_kumasaca
Gelidocalamus_tessellatus
Ferrocalamus_rimosivaginus
Indocalamus_wilsonii
Ampelocalamus_calcareus
Otatea_glauca
Otatea_acuminata
Olmeca_reflexa
Guadua_weberbaueri
Rhipidocladum_pittieri
Chusquea_circinata
Chusquea_liebmannii
Chusquea_sp.
Chusquea_spectabilis
Bambusa_oldhamii
Bambusa_multiplex
Bambusa_emeiensis
Bambusa_arnhemica
Bambusa_bambos
Dendrocalamus_latiflorus
Greslania_sp.
Neololeba_atra
Hickelia_madagascariensis
Lithachne_pauciflora
Cryptochloa_strictiflora
Olyra_latifolia
Raddia_brasiliensis
Diandrolyra_sp.
Pariana_campestris
Pariana_sp.
Pariana_radiciflora
Eremitis_sp.
Buergersiochloa_bambusoides
Oryza_sativa_Indica
Oryza_nivara
Oryza_rufipogon
Oryza_barthii
Oryza_glaberrima
Oryza_meridionalis
Oryza_glumipatula
Oryza_longistaminata
Oryza_punctata
Oryza_officinalis
Oryza_australiensis
Prosphytochloa_prehensilis
Leersia_tisserantii
Zizania_aquatica
Rhynchoryza_subulata
Chikusichloa_aquatica
Potamophila_parviflora
Humbertochloa_bambusiuscula
Ehrharta_bulbosa
Microlaena_stipoides
Streptogyna_americana
Sorghum_timorense
Sorghum_bicolor
Saccharum_hybrid_cv._NCo_310
Imperata_cylindrica
Sorghastrum_nutans
Eulalia_aurea
Bothriochloa_alta
Capillipedium_venustrum
Iseilema_macratherum
Themeda_sp.
Hyparrhenia_subplumosa
Dheteropogon_amplectans_var._catangensis
Rottboellia_cochinchinensis
Coix_lacryma-jobi
Ischaemum_afrum
Zea_mays_subsp._mays
Arthraxon_prionodes
Arundinella_deppeana
Steinchisma_laxum
Otachyrium_versicolor
Plagiantha_tenella
Oncorachis_ramosa
Coleataenia_prionitis
Paspalum_fimbriatum
Paspalum_dilatatum
Paspalum_glazovii
Axonopus_fissifolius
Setaria_italica
Setaria_viridis
Cenchrus_americanus
Setaria_geminata
Urochloa_reptans
Eriochloa_meyeriana
Megathrysus_maximus
Whiteochloa_capillipes
Panicum_capillare
Panicum_virgatum
Dichanthelium_acuminatum
Thyridolepis_xerophila
Alloteropsis_semialata
Alloteropsis_angusta
Alloteropsis_cimicina
Amphicarpum_muhlenbergianum
Oplismenus_hirtellus
Echinochloa_oryzicola
Echinochloa_crus-galli
Digitaria_exilis
Lecomtella_madagascariensis
Zeugites_pittieri
Chasmanthium_sessiliflorum
Loudetiopsis_kerstingii
Danthoniopsis_dinteri
Thysanolaena_latifolia
Centotheca_lappacea
Eustachys_glauca
Oxychloris_scariosa
Chloris_barbata
Leptochloa_plurriflora
Astrebla_pectinata
Dactyloctenium_aegyptium
Trichoneura_grandiglumis
Triodia_stipoides
Triodia_wiseana
Melanocenchris_abyssinica
Halopyrum_mucronatum
Distichlis_bajaensis
Distichlis_spicata
Bouteloua_curtipendula
Bouteloua_gracilis
Hilaria_cenchroides
Hilaria_rigida
Sporobolus_maritimus
Sporobolus_michauxianus
Sporobolus_heterolepis
Zoysia_macrantha
Eragrostis_minor
Eragrostis_tef
Uniola_paniculata
Neyraudia_reynaudiana
Centropodia_glauca
Rytidosperma_pallidum
Rytidosperma_semiannulare
Tenaxia_guillarmodiae
Tribolium_hispidum
Danthonia_californica
Chaetobromus_involucratus_subsp._dregeanus
Chionochloa_macra
Phragmites_australis
Hakonechloa_macra
Elytrophorus_spicatus
Monachather_paradoxa
Isachne_distichophylla
Eriachne_stipacea
Micraira_spiciformis
Stipagrostis_hirtigluma
Stipagrostis_uniplumis_var._uniplumis
Sartidia_dewinteri
Aristida_purpurea
Aristida_ternipes
Puelia_olyriformis
Leptaspis_zeylanica
Leptaspis_banksii
Pharus_latifolius
Pharus_lappulaceus
Anomochloa_marantoidea
Streptochaeta_spicata
0.01

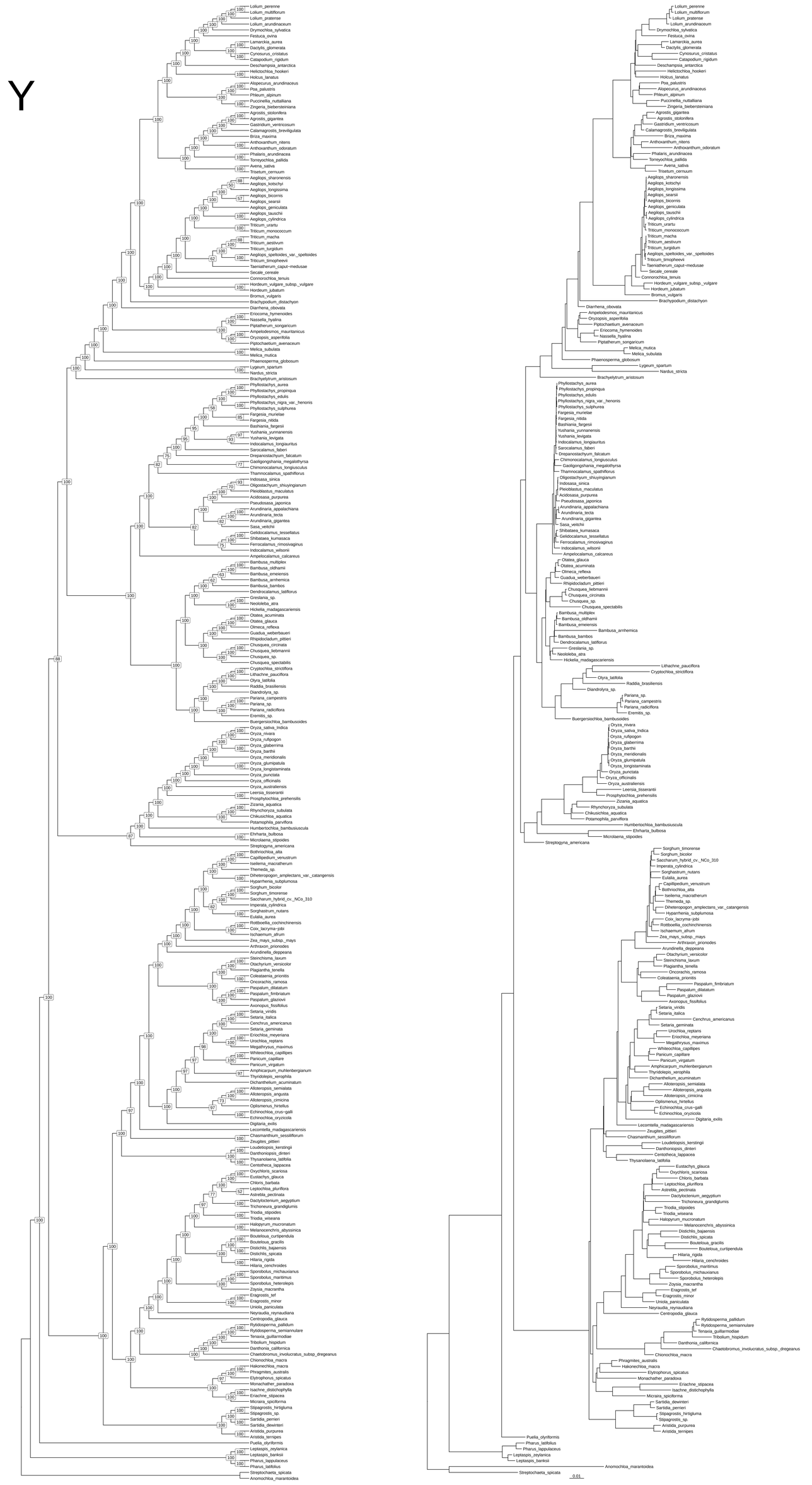
Y
Lolium_perenne
Lolium_multiflorum
Lolium_pratense
Lolium_arundinaceum
Drymochloa_sylvatica
Festuca_ovina
Lamarckia_aurea
Dactylis_glomerata
Cynosurus_cristatus
Catapodium_rigidum
Deschampsia_antarctica
Helictochloa_hookeri
Holcus_lanatus
Alopecurus_arundinaceus
Poa_palustris
Phleum_alpinum
Puccinellia_nuttalliana
Zingeria_biebersteiniana
Agrostis_stolonifera
Agrostis_gigantea
Gastridium_ventricosum
Calamagrostis_brevilgulata
Briza_maxima
Anthoxanthum_nitens
Anthoxanthum_odoratum
Phalaris_arundinacea
Torreyochloa_pallida
Avena_sativa
Trisetum_cernuum
Aegilops_sharonensis
Aegilops_kotschyi
Aegilops_longissima
Aegilops_bicornis
Aegilops_searsii
Aegilops_geniculata
Aegilops_tauschii
Aegilops_cylindrica
Triticum_urartu
Triticum_monococcum
Triticum_macha
Triticum_aestivum
Triticum_turgidum
Aegilops_speltoides_var._speltoides
Triticum_timopheevii
Taeniatherum_caput-medusae
Secale_cereale
Connorochloa_tenuis
Hordeum_vulgare_subsp._vulgare
Hordeum_jubatum
Bromus_vulgaris
Brachypodium_distachyon
Diarrhena_obovata
Eriocoma_hymenoides
Nassella_hyalina
Piptatherum_songaricum
Ampelodesmos_mauritanicus
Oryzopsis_asperifolia
Piptochaetium_avenaceum
Melica_subulata
Melica_mutica
Phaenosperma_globosum
Lygeum_spartum
Nardus_stricta
Brachyelytrum_aristosum
Phyllostachys_aurea
Phyllostachys_propinqua
Phyllostachys_edulis
Phyllostachys_nigra_var._henonis
Phyllostachys_sulphurea
Fargesia_murielae
Fargesia_nitida
Bashania_fargesii
Yushania_yunnanensis
Yushania_levigata
Indocalamus_longiauritus
Sarocalamus_faberi
Drepanostachyum_falcatum
Gaoligongshania_megalothyrsa
Chimonocalamus_longiusculus
Thamnocalamus_spathiflorus
Indosasa_sinica
Oligostachyum_shiuyinglanum
Pleioblastus_maculatus
Acidosasa_purpurea
Pseudosasa_japonica
Arundinaria_appalachiana
Arundinaria_tecta
Arundinaria_gigantea
Sasa_veitchii
Shibataea_kumasaca
Gelidocalamus_tessellatus
Ferrocalamus_rimosivaginus
Indocalamus_wilsonii
Ampelocalamus_calcareus
Bambusa_multiplex
Bambusa_oldhamii
Bambusa_emeiensis
Bambusa_arnhemica
Bambusa_bambos
Dendrocalamus_latiflorus
Greslania_sp.
Neololeba_atra
Hickelia_madagascariensis
Otatea_acuminata
Otatea_glauca
Olmeca_reflexa
Guadua_weberbaueri
Rhipidocladum_pittieri
Chusquea_circinata
Chusquea_liebmannii
Chusquea_sp.
Chusquea_spectabilis
Cryptochloa_strictiflora
Lithachne_pauciflora
Olyra_latifolia
Raddia_brasiliensis
Diandrolyra_sp.
Pariana_campestris
Pariana_sp.
Pariana_radiciflora
Eremitis_sp.
Buergersiochloa_bambusoides
Oryza_sativa_indica
Oryza_nivara
Oryza_rufipogon
Oryza_glaberrima
Oryza_barthii
Oryza_meridionalis
Oryza_glumipatula
Oryza_longistaminata
Oryza_punctata
Oryza_officinalis
Oryza_australiensis
Leersia_tisserantii
Prosphytochloa_prehensilis
Zizania_aquatica
Rhynchoryza_subulata
Chikusichloa_aquatica
Potamophila_parviflora
Humbertochloa_bambusiuscula
Ehrharta_bulbosa
Microlaena_stipoides
Streptogyna_americana
Bothriochloa_alta
Capillipedium_venustrum
Iseilema_macratherum
Themeda_sp.
Diheteropogon_amplectans_var._catangensis
Hyparrhenia_subplumosa
Sorghum_bicolor
Sorghum_timorense
Saccharum_hybrid_cv._NCo_310
Imperata_cylindrica
Sorghastrum_nutans
Eulalia_aurea
Rottboellia_cochinchinensis
Coix_lacryma-jobi
Ischaemum_afrum
Zea_mays_subsp._mays
Arthraxon_prionodes
Arundinella_deppeana
Steinchisma_laxum
Otachyrium_versicolor
Plagiantha_tenella
Coleataenia_prionitis
Oncorachis_ramosa
Paspalum_dilatatum
Paspalum_fimbriatum
Paspalum_glazovii
Axonopus_fissifolius
Setaria_viridis
Setaria_italica
Cenchrus_americanus
Setaria_geminata
Eriochloa_meyeriana
Urochloa_reptans
Megathrysus_maximus
Whiteochloa_capillipes
Panicum_capillare
Panicum_virgatum
Amphicarpum_muhlenbergianum
Thyridolepis_xerophila
Dichanthelium_acuminatum
Alloteropsis_semialata
Alloteropsis_angusta
Alloteropsis_cimicina
Oplismenus_hirtellus
Echinochloa_crus-galli
Echinochloa_oryzicola
Digitaria_exilis
Lecomtella_madagascariensis
Zeugites_pittieri
Chasmanthium_sessiliflorum
Loudetopsis_kerstingii
Danthoniopsis_dinteri
Centotheca_lappacea
Thysanolaena_latifolia
Eustachys_glauca
Oxychloris_scariosa
Chloris_barbata
Leptochloa_plurriflora
Astrebla_pectinata
Dactyloctenium_aegyptium
Trichoneura_grandiglumis
Triodia_stipoides
Triodia_wiseana
Halopyrum_mucronatum
Melanocenchris_abyssinica
Bouteloua_curtipendula
Bouteloua_gracilis
Distichlis_bajaensis
Distichlis_spicata
Hilaria_rigida
Hilaria_cenchroides
Sporobolus_michauxianus
Sporobolus_maritimus
Sporobolus_heterolepis
Zoysia_macrantha
Eragrostis_tef
Eragrostis_minor
Uniola_paniculata
Neyraudia_reynaudiana
Centropodia_glauca
Rytidosperma_pallidum
Rytidosperma_semiannulare
Tenaxia_guillarmodiae
Tribolium_hispidum
Danthonia_californica
Chaetobromus_involucratus_subsp._dregeanus
Chionochloa_macra
Hakonechloa_macra
Phragmites_australis
Elytrophorus_spicatus
Monachather_paradoxa
Isachne_distichophylla
Eriachne_stipacea
Micraira_spiciformis
Stipagrostis_hirtigluma
Stipagrostis_sp.
Sartidia_perrieri
Sartidia_dewinteri
Aristida_purpurea
Aristida_ternipes
Puelia_olyriformis
Leptaspis_zeylanica
Leptaspis_banksii
Pharus_latifolius
Pharus_lappulaceus
Streptochaeta_spicata
Anomochloa_marantoidea
0.01

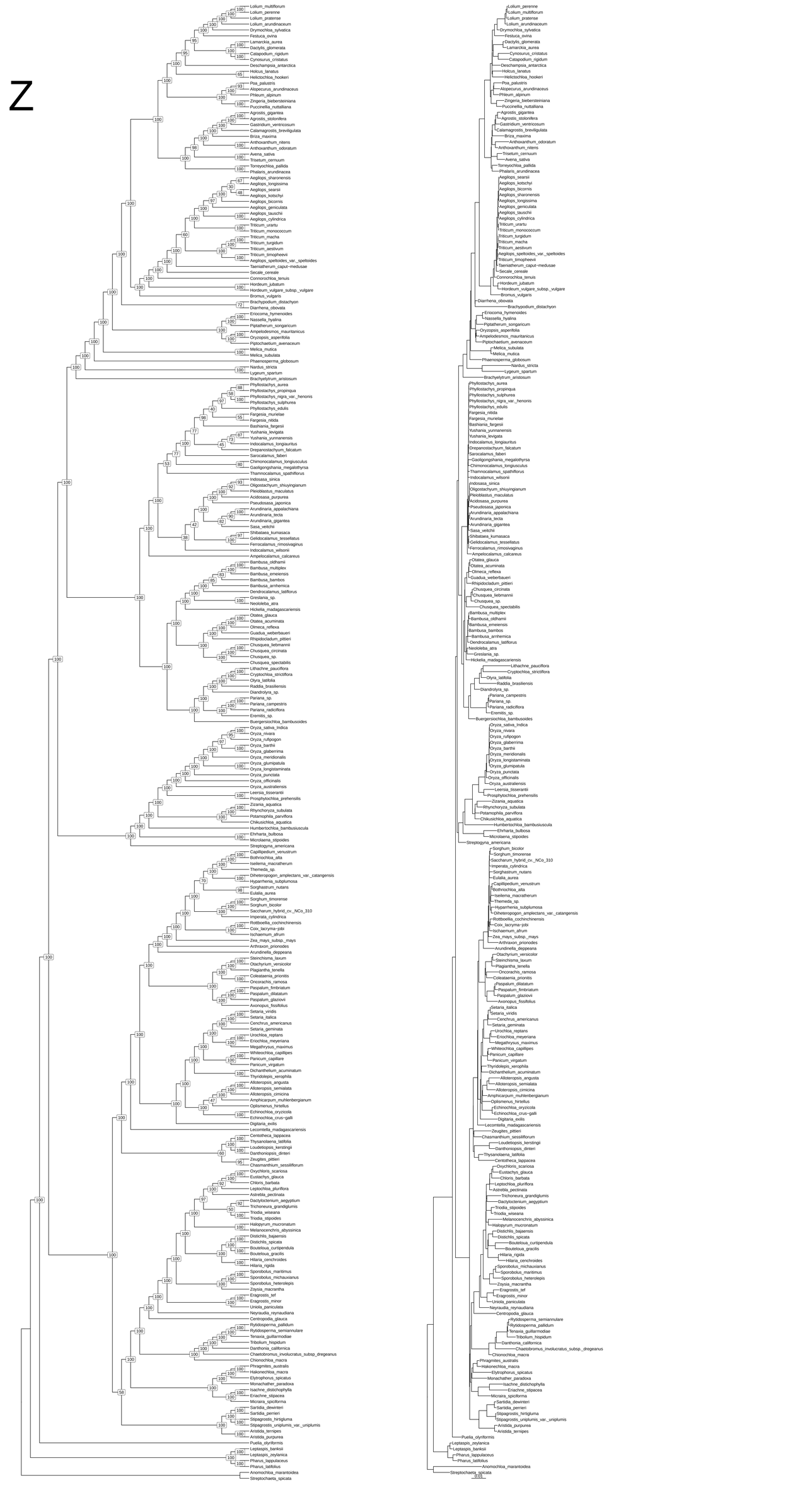
Z
Lolium_multiflorum
Lolium_perenne
Lolium_pratense
Lolium_arundinaceum
Drymochloa_sylvatica
Festuca_ovina
Lamarckia_aurea
Dactylis_glomerata
Catapodium_rigidum
Cynosurus_cristatus
Deschampsia_antarctica
Holcus_lanatus
Helictochloa_hookeri
Poa_palustris
Alopecurus_arundinaceus
Phleum_alpinum
Zingeria_biebersteiniana
Puccinellia_nuttalliana
Agrostis_gigantea
Agrostis_stolonifera
Gastridium_ventricosum
Calamagrostis_breviligulata
Briza_maxima
Anthoxanthum_nitens
Anthoxanthum_odoratum
Avena_sativa
Trisetum_cernuum
Torreyochloa_pallida
Phalaris_arundinacea
Aegilops_sharonensis
Aegilops_longissima
Aegilops_searsii
Aegilops_kotschyi
Aegilops_bicornis
Aegilops_geniculata
Aegilops_tauschii
Aegilops_cylindrica
Triticum_urartu
Triticum_monococcum
Triticum_macha
Triticum_turgidum
Triticum_aestivum
Triticum_timopheevii
Aegilops_speltoides_var._speltoides
Taeniatherum_caput-medusae
Secale_cereale
Connorochloa_tenuis
Hordeum_jubatum
Hordeum_vulgare_subsp._vulgare
Bromus_vulgaris
Brachypodium_distachyon
Diarrhena_obovata
Eriocoma_hymenoides
Nassella_hyalina
Piptatherum_songaricum
Ampelodesmos_mauritanicus
Oryzopsis_asperifolia
Piptochaetium_avenaceum
Melica_mutica
Melica_subulata
Phaenosperma_globosum
Nardus_stricta
Lygeum_spartum
Brachyelytrum_aristosum
Phyllostachys_aurea
Phyllostachys_propinqua
Phyllostachys_nigra_var._henonis
Phyllostachys_sulphurea
Phyllostachys_edulis
Fargesia_nitida
Fargesia_murielae
Bashania_fargesii
Yushania_yunnanensis
Yushania_levigata
Indocalamus_longiauritus
Drepanostachyum_falcatum
Sarocalamus_faberi
Gaoligongshania_megalothyrsa
Chimonocalamus_longiusculus
Thamnocalamus_spathiflorus
Indocalamus_wilsonii
Indosasa_sinica
Oligostachyum_shiuyingianum
Pleioblastus_maculatus
Acidosasa_purpurea
Pseudosasa_japonica
Arundinaria_appalachiana
Arundinaria_tecta
Arundinaria_gigantea
Sasa_veitchii
Shibataea_kumasaca
Gelidocalamus_tessellatus
Ferrocalamus_rimosivaginus
Ampelocalamus_calcareus
Otatea_glauca
Otatea_acuminata
Olmeca_reflexa
Guadua_weberbaueri
Rhipidocladum_pittieri
Chusquea_circinata
Chusquea_liebmannii
Chusquea_sp.
Chusquea_spectabilis
Bambusa_multiplex
Bambusa_oldhamii
Bambusa_emeiensis
Bambusa_bambos
Bambusa_arnhemica
Dendrocalamus_latiflorus
Neololeba_atra
Greslania_sp.
Hickelia_madagascariensis
Lithachne_pauciflora
Cryptochloa_strictiflora
Olyra_latifolia
Raddia_brasiliensis
Diandrolyra_sp.
Pariana_campestris
Pariana_sp.
Pariana_radiciflora
Eremitis_sp.
Buergersiochloa_bambusoides
Oryza_sativa_Indica
Oryza_rivara
Oryza_rufipogon
Oryza_glaberrima
Oryza_barthii
Oryza_meridionalis
Oryza_longistaminata
Oryza_glumipatula
Oryza_punctata
Oryza_officinalis
Oryza_australiensis
Leersia_tisserantii
Prosphytochloa_prehensilis
Zizania_aquatica
Rhynchoryza_subulata
Potamophila_parviflora
Chikusichloa_aquatica
Humbertochloa_bambusiuscula
Ehrharta_bulbosa
Microlaena_stipoides
Streptogyna_americana
Sorghum_bicolor
Sorghum_timorense
Saccharum_hybrid_cv._NCo_310
Imperata_cylindrica
Sorghastrum_nutans
Eulalia_aurea
Capillipedium_venustrum
Bothriochloa_alta
Iseilema_macratherum
Themeda_sp.
Hyparrhenia_subplumosa
Diheteropogon_amplectans_var._catangensis
Rottboellia_cochinchinensis
Coix_lacryma-jobi
Ischaemum_afrum
Zea_mays_subsp._mays
Arthraxon_prionodes
Arundinella_deppeana
Otachyrium_versicolor
Steinchisma_laxum
Plagiantha_tenella
Oncorachis_ramosa
Coleataenia_prionitis
Paspalum_dilatatum
Paspalum_fimbriatum
Paspalum_glazovii
Axonopus_fissifolius
Setaria_italica
Setaria_viridis
Cenchrus_americanus
Setaria_geminata
Urochloa_reptans
Eriochloa_meyeriana
Megathrysus_maximus
Whiteochloa_capillipes
Panicum_capillare
Panicum_virgatum
Thyridolepis_xerophila
Dichanthelium_acuminatum
Alloteropsis_angusta
Alloteropsis_semialata
Alloteropsis_cimicina
Amphicarpum_muhlenbergianum
Oplismenus_hirtellus
Echinochloa_oryzicola
Echinochloa_crus-galli
Digitaria_exilis
Lecomtella_madagascariensis
Cenotheca_lappacea
Thysanolaena_latifolia
Loudetopsis_kerstingii
Danthoniopsis_dinteri
Zeugites_pittieri
Chasmanthium_sessiliflorum
Oxychloris_scariosa
Eustachys_glauca
Chloris_barbata
Leptochloa_pluriflora
Astrebla_pectinata
Dactyloctenium_aegyptium
Trichoneura_grandiglumis
Triodia_wiseana
Triodia_stipoides
Halopyrum_mucronatum
Melanocenchris_abyssinica
Distichlis_bajaensis
Distichlis_spicata
Bouteloua_curtipendula
Bouteloua_gracilis
Hilaria_cenchroides
Hilaria_rigida
Sporobolus_maritimus
Sporobolus_michauxianus
Sporobolus_heterolepis
Zoysia_macrantha
Eragrostis_tef
Eragrostis_minor
Uniola_paniculata
Neyraudia_reynaudiana
Centropodia_glauca
Rytidosperma_pallidum
Rytidosperma_semiannulare
Tenaxia_guillarmodiae
Tribolium_hispidum
Danthonia_californica
Chaetobromus_involucratus_subsp._dregeanus
Chionochloa_macra
Phragmites_australis
Hakonechloa_macra
Elytrophorus_spicatus
Monachather_paradoxa
Isachne_distichophylla
Eriachne_stipacea
Micraira_spiciformis
Sartidia_dewinteri
Sartidia_perrieri
Stipagrostis_hirtigluma
Stipagrostis_uniplumis_var._uniplumis
Aristida_purpurea
Aristida_ternipes
Puelia_olyriformis
Leptaspis_zeylanica
Leptaspis_banksii
Pharus_lappulaceus
Pharus_latifolius
Anomochloa_marantoidea
Streptochaeta_spicata
0.01
